# Neonatal bone marrow interstitial fluid supports expansion and osteogenic ability of human bone marrow mesenchymal stromal cells

**DOI:** 10.1038/s41413-025-00496-z

**Published:** 2025-12-15

**Authors:** Junfeng Zhang, Tengjiao Ma, Lan Ke, Huan Zhang, Jiaxin Hu, Shunping Li, Hailong Wang, Anming Meng

**Affiliations:** 1 Guangzhou National Laboratory, Guangzhou International Bio Island, Guangzhou, Guangdong Province China; 2 Laboratory of Molecular Developmental Biology, State Key Laboratory of Membrane Biology, Tsinghua-Peking Center for Life Sciences, School of Life Sciences, Tsinghua University, Beijing, China

**Keywords:** Bone, Bone quality and biomechanics

## Abstract

The bone marrow microenvironment is critical for the maintenance and functionality of stem/progenitor cells, which are essential for bone development and regeneration. However, the composition and potential use of bone marrow interstitial fluid have not been well explored. In this study, we report the role of neonatal bovine bone marrow interstitial fluid (NBIF) in enhancing the bone regeneration capacity of human bone marrow mesenchymal stem cells (hBMSCs). Unlike adult bovine bone marrow interstitial fluid (ABIF), NBIF-fed hBMSCs exhibit enhanced self-renewal and osteogenic potential and bone marrow homing ability, along with transcriptome changes as compared to hBMSCs cultured in standard fetal bovine serum (FBS) supplemented medium. Mass spectrometry analysis reveals that multiple secreted factors associated with tissue repair and bone development are enriched in NBIF compared to FBS and ABIF. The combined use of NBIF-enriched Nerve Growth Factor (NGF), Lactoferrin (LTF), and High Mobility Group Protein B1 (HMGB1), together with Insulin-Like Growth Factor 1(IGF1) for culturing hBMSCs in the presence of FBS can enhance osteogenic potential and bone marrow homing ability, mimicking NBIF’s effects. These findings highlight the role of interstitial fluid in the bone marrow microenvironment and its potential to optimize stem cell-based therapies.

## Introduction

The interstitial fluid within a tissue or organ fills the spaces between cells and is rich in nutrients, hormones, cytokines, and signaling molecules. It has been shown to be crucial in tissue homeostasis and disease progression.^1–5^ For instance, myokines and adipokines derived from interstitial fluid are important for the body’s energy balance.^6^

The bone marrow (BM) is a soft, spongy tissue within the cavities of the mammalian body’s bones. The BM harbors many types of cells, including hematopoietic stem cells (HSCs) and their derivatives, mesenchymal stromal cells (MSCs), adipocytes, osteoblasts, osteoclasts, endothelial cells, and innervated nerves. The cellular composition and biological/pathological implications of BM changes with developmental stages.^7–14^ The BM in infants and children, referred to as red BM, harbors rich HSCs, which upon differentiation generate large amounts of red blood cells and immune cells to support rapid growth, immune system development and increased oxygen demand, and MSCs that mainly differentiate into osteoblasts. In adults, the BM, now looking yellow in color, contains more adipocytes differentiated from MSCs and more dysfunctional HSCs, acting as an energy storage source. It is obvious that the composition of BM interstitial fluid would also change with age due to changing cellular composition. However, it remains elusive how the BM interstitial fluid changes dynamically.

The BM-derived MSCs (BMSCs) have been considered as an important source for stem cell based therapies, due to its remarkable immunomodulatory properties, osteogenic capacity, and homing ability relying on C-X-C Motif Chemokine Ligand 12 (CXCL12)/ C-X-C Motif Chemokine Receptor 4 (CXCR4) axis.^15^ Like other sources of MSCs, BMSCs after several passages in vitro show decreased pluripotency and proliferation, which are mainly driven by replicative senescence, telomere shortening, oxidative stress, and epigenetic changes.^16–19^ In addition, the expression of CXCR4 receptor in BMSCs rapidly decreases upon short-term in vitro expansion.^20,21^ These alterations could limit the therapeutic application of BMSCs.

We assume that the BM interstitial fluid in the newborn animals contains important niche factors to support BMSC proliferation, to sustain their pluripotency, and to bias their differentiation potential. In this study, therefore, we used neonatal bovine BM interstitial fluid (NBIF) to culture hBMSCs and characterized their molecular changes and differentiation capacity. We also analyzed the proteomic composition of NBIF and identified several NBIF factors that help improve the quality of cultured hBMSCs.

## Results

### NBIF supports hBMSC expansion with sustained specific surface markers

**Fig. 1:**
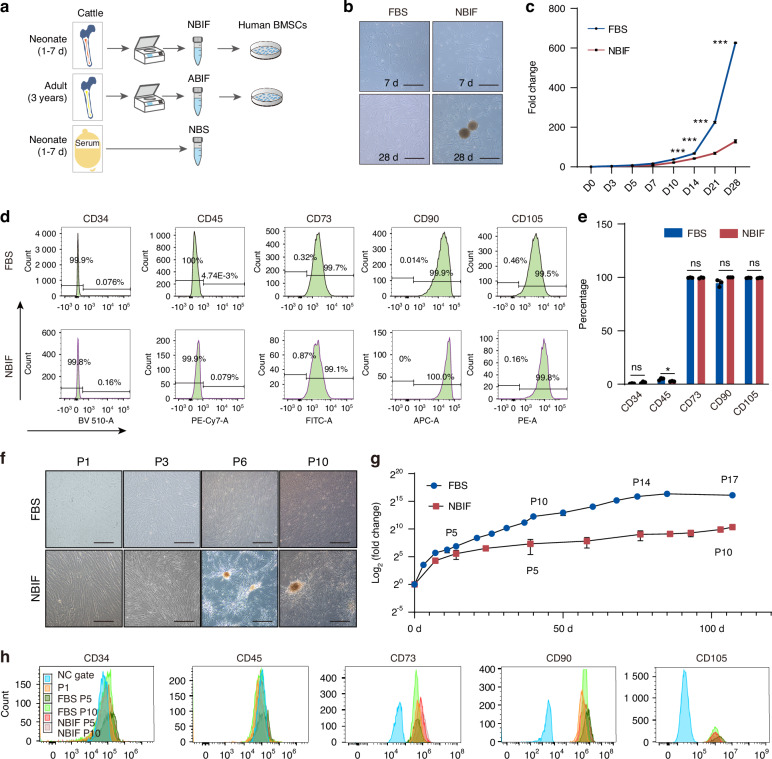
The effects of NBIF, ABIF and FBS on in vitro cultured hBMSCs. **a** Scheme of experimental procedures. **b** Morphology of hBMSCs in vitro cultured for 7 days (D7) or 28 days (D28) under indicated culture conditions. Note the formation of spheroid-like structures in NBIF culture condition. Scale bars, 100 μm. **c** Growth curve of hBMSCs cultured under different conditions. 1 × 10^5^ hBMSCs were seeded in a 6-cm² dish. After reaching 80% confluence, cells were re-plated for expansion at a density of 3 000 cells/cm². The expansion fold change between MSCs in NBIF medium and fetal bovine serum (FBS) medium was expressed as the mean of three experiments from day zero (D0) to day 28 (D28). Data were expressed as mean ± standard error of the mean (SEM) of the fold change across three replicates for each group. *P*-values were obtained from an unpaired *t*-test; **P ≤ 0.01; ***P ≤ 0.001. **d** Immunophenotype analysis of hBMSCs after 14-day culture by flow cytometry using specific surface markers. The results shown were representative results. n.s: not significant. **e** Statistical data of flow cytometry. The percentage of positive cells was obtained from three independent experiments, expressed as mean ± standard error of the mean (SEM) of the percentages across three replicates for each group. *P*-values from the unpaired *t*-test; ns, not significant; *P ≤ 0.05. **f–h** Morphology (**f**), growth curve (**g**) and flow cytometry result (**h**) of hBMSCs after long-term culture. P, passage. Scale bar, 100 μm

We hypothesized that neonatal BM interstitial fluid contains rich substances, including growth factors, to support the proliferation of stem cells and progenitor cells. To obtain a large quantity, we decided to isolate interstitial fluid from long bones of neonatal (1–7 days old) and adult (around 3 years old) cattle (Fig. 1a) for testing effects on hBMSCs proliferation and differentiation. By optimizing previous methods for interstitial fluid isolation,^6,22^ we were able to harvest approximately 206 ± 13 mL or 563 ± 26 mL of purified BIF from each neonatal or adult cattle, respectively, which was diluted to have an equal concentration of 0.25 mg/mL protein as stocks.

The hBMSCs were cultured in the DMEM/F-12 basal medium supplemented with equal amounts of protein-containing neonatal BIF (NBIF), adult BIF (ABIF), or commercial fetal bovine serum (FBS). Unlike those cultured in NBIF or FBS-supplied medium, hBMSCs or other types of human MSCs in ABIF medium exhibited significantly reduced proliferation (Fig. S1a, b), and ABIF-fed hBMSCs underwent significant senescence as early as passage 2 (Fig. S1c and d). We assume the much slower growth in the ABIF group suggests a deficiency in essential growth factors and nutrients, which is supported by the significant reduction in SA-β-gal-positive cells when adding 5% FBS to the medium supplement (Fig. S1e). Therefore, our subsequent analyses were focused on NBIF and FBS. Our initial observation indicated that NBIF was able to promote hBMSC expansion during a 4-week culture period, albeit at a lower capacity than FBS (Fig. 1b, c), and NBIF-cultured hBMSCs retained the BMSC-specific markers CD73, CD90 or CD105 and were negative for the hematopoietic markers CD34 and CD45 as examined by flow cytometry at 2 weeks (Fig. 1d, e). Even after 10 passages over 100 days of continuous culture, hBMSCs in NBIF were still proliferative and expressed BMSC-specific markers (Fig. 1f–h). To assess genomic stability, karyotyping of NBIF-cultured hBMSCs at P8 revealed no detectable chromosomal abnormalities (Fig. S2a). However, there is significant upregulation of cell cycle inhibitors, mainly *P21 (CDKN1A)* and *P16* (*CDKN2A*) (Fig. S2b), as well as an increasing number of SA-β-gal^+^ cells, both in FBS-fed and NBIF-fed BMSCs (Fig. S2c, d) at passage 10. These data suggest that while NBIF effectively preserves the identity and genomic stability of hBMSCs during long-term culture, it does not prevent the replicative senescence, which is similar to FBS. Interestingly, NBIF-nourished hBMSCs usually formed some spheroid lumps after 3-4 weeks (Fig. 1b, f) for unknown reasons. These observations together imply that, like FBS, NBIF possesses bioactivity to maintain hBMSC identity.

### NBIF-fed hBMSCs have greater osteogenic differentiation capacity with distinct molecular changes

**Fig. 2:**
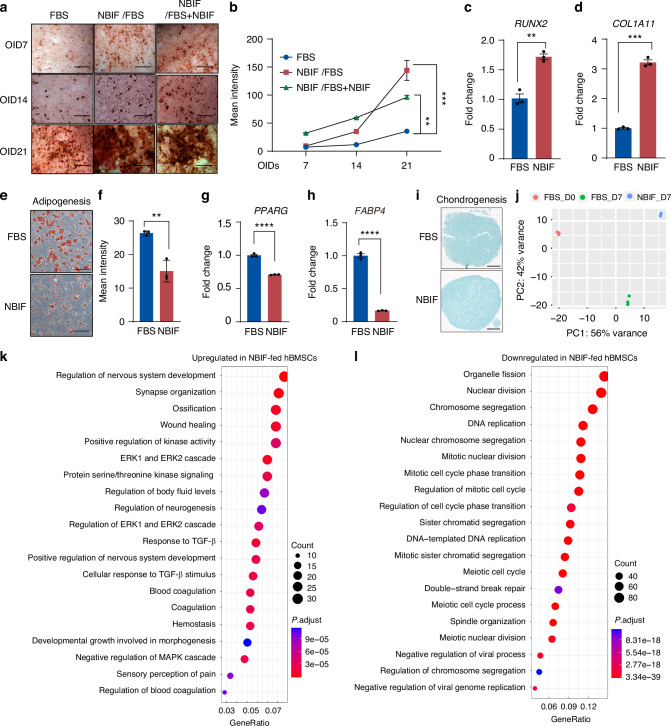
NBIF-fed hBMSCs have greater osteogenic differentiation capacity with distinct molecular changes. **a, b** Representative osteogenic induction assay images (**a**) and quantification results (**b**) of hBMSCs. The BMSC were first cultured in FBS- (column 1) or NBIF- (columns 2 and 3) supplemented medium for 7 days and then subjected to osteogenic induction (OI) for 7, 14 and 21 days in the presence of FBS (columns 1 and 2) or FBS plus NBIF (column 3), followed by Alizarin Red staining. Data in (**b**) [as well as in (**c**, **d**, **f**–**h**)] were expressed as mean ± standard error of the mean (SEM) of the fold change across three replicates for each group. *P*-values were obtained from an unpaired *t*-test; **P ≤ 0.01, ***P ≤ 0.001. Scale bar = 100 μm. **c**, **d** RT-PCR detection of expression levels of the osteogenesis-related genes *RUNX2* (**c**) and *COL1A1* (**d**) after 28-day osteogenic induction. **e**, **f** Representative images and quantification data of adipogenic differentiation of hBMSCs. The hBMSCs were first cultured in FBS or NBIF-supplemented medium for 7 days and then in adipogenic differentiation medium for 28 days, followed by Oil red staining. Scale bar = 100 μm. **g, h** RT-PCR detection of expression levels of the adipogenesis-related genes *PPARG* (**g**) and *FABP4* (**h**) after 28-day of adipogenic induction. **i** Represent images of Alcian Blue staining to indicate Chondrogenesis. **j** Unsupervised principal component analysis (PCA) of RNA-seq data based on gene transcription profiles from the indicated hBMSC samples. MSCs cultured in NBIF and those cultured in FBS distinctly clustered within the second principal component (PC2), which explained 42% of the observed variation. **k, l** Gene Ontology (GO) analysis for upregulated (**k**) and downregulated (**l**) genes in hBMSCs cultured in NBIF-supplied medium compared to FBS-supplied medium. The top 20 enriched biological processes were presented. Scale bar = 500 μm

MSCs possess multipotency, usually delineated by their differentiation capacity into osteoblasts, adipocytes, and chondrocytes. We investigated the osteogenic capacity of hBMSCs after culturing for 7 days in the basal medium with the addition of NBIF or FBS, followed by osteogenic induction in the medium with FBS only or with FBS plus NBIF for different durations. Alizarin red staining after osteogenic induction revealed significantly more calcium deposition in NBIF-nourished hBMSCs compared to FBS-fed hBMSCs (Fig. 2a, b). RT-PCR analysis showed that the osteogenic markers *RUNX2* and *COL1A1* were significantly upregulated after 7-day culture and 21-day osteogenic induction in the presence of NBIF (Fig. 2c, d). Notably, the NBIF-cultured hBMSCs exhibited more significant osteogenic differentiation than both FBS-cultured cells at Day 7 and the parental (Day 0) cells from which they were derived, suggesting that the enhanced osteogenic potential of NBIF-fed MSC is a direct effect of bioactive components within NBIF, but not primarily a trade-off between expansion and differentiation (Fig. S3a, b). Conversely, NBIF treatment reduced hBMSC adipogenic differentiation capacity, as evidenced by decreased Oil Red staining signals (Fig. 2e, f) and downregulated expression of the adipogenic markers *PPARG* and *FABP4* (Fig. 2g, h). Alcian blue staining indicated that the presence of NBIF during hBMSC culturing and chondrogenic induction had no effect on chondrogenic differentiation capacity (Fig. 2i). These observations suggest that, compared to FBS, NBIF can enhance hBMSCs osteogenic differentiation potential with reduced adipogenic capacity.

To understand molecular changes after culture in hBMSCs, we performed transcriptome analysis of hBMSCs after 7-day culture in NBIF or FBS. Principal Component Analysis (PCA) demonstrated a clear separation between NBIF- and FBS-fed hBMSCs (Fig. 2j), with a total of 1 614 differentially expressed genes identified, including 890 upregulated and 724 downregulated genes in the NBIF condition. The upregulated genes were linked to development and tissue repair processes, including neural development and bone formation (Fig. 2k). In contrast, most downregulated genes were associated with DNA replication and cell proliferation, consistent with the observed slower proliferation rate (Fig. 2l). Interestingly, interferon and inflammation signaling pathways (Fig. 2l), as well as major MHC Class I and II genes such as *HLA-A*, *HLA-B*, *HLA-C*, *HLA-DRA*, and *HLA-DRB1* (Fig. S4a–c) were downregulated in NBIF-fed hBMSCs, suggesting a reduced immunogenicity.

It is believed that MSCs can actively regulate the microenvironment by producing cytokines and growth factors.^23,24^ Gene Set Enrichment Analysis (GSEA) of the NBIF-fed hBMSCs transcriptome revealed significant enrichment of protein secretion (Fig. S5a) and VEGF signaling pathways (Fig. S5b), suggesting that NBIF in the medium may enhance production of secretory factors. To verify this idea, we performed a multiplex Luminex analysis of culture supernatants of hBMSCs, which were collected after removal of the original culture medium and an additional 24-hour culture in fresh DMEM/F-12 containing 1% FBS. Results revealed that among the 48 detected factors, 7 were significantly decreased and 17 were significantly increased in NBIF-fed hBMSCs compared to FBS-fed hBMSCs (Fig. S5c for examples and Table S1 for the full list of factors). Importantly, Leukemia Inhibitory Factor (LIF) and Platelet-derived Growth Factor (PDGF) were maintained at comparable levels between the two groups, while the VEGF secretion from NBIF-fed hBMSCs dramatically decreased compared to FBS-fed hBMSCs. Conversely, tissue repair-related factors, including Fibroblast Growth Factor 2 (FGF2), Hepatocyte Growth Factor (HGF), and CXCL12 showed significant increases. Interestingly, Interleukin 1 Receptor Antagonist (IL-1RA), which is associated with immunomodulatory functions,^25^ was also slightly elevated. Our data support the idea that hBMSCs may produce different types and amounts of secreted factors depending on culture nutrition.

### NBIF helps maintain BMSCs’ self-renewal and bone marrow homing ability

**Fig. 3:**
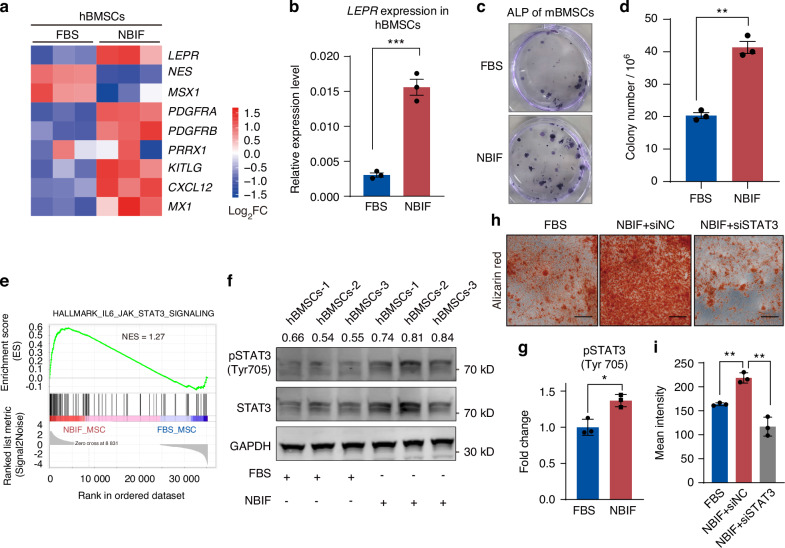
NBIF helps maintain hBMSCs self-renewal ability. **a** Heatmap displaying relative expression levels of skeletal progenitor-related genes in hBMSCs cultured in different conditions. The transcriptome data were used. **b** *LEPR* relative expression levels (normalized to *GAPDH*) of hBMSCs, evaluated by quantitative RT-PCR. Data [and also in (**d**) and (**h**)] were expressed as mean ± standard error of the mean (SEM) of the fold change across three replicates for each group. *P*-values were obtained from an unpaired *t*-test; **P ≤ 0.01, ***P ≤ 0.001. **c, d** Representative images (**c**) and quantification data (**d**) of alkaline phosphatase-positive hBMSC CFUs. CFU formation from hBMSCs was analyzed at passage 1 after 14 days of culture, followed by the alkaline phosphatase activity assay. **e** Gene set enrichment analysis (GSEA) using the transcriptome data indicates that the IL6_JAK_STAT3 signaling pathway is enriched in NBIF-supplied hBMSCs with FDR < 0.25. **f**, **g** Western blot detection of phospho-STAT3(Tyr705) level in BMSCs cultured in different conditions for 7 days. Three replicate were shown for each condition, relative phospho-STAT3(Tyr705) level were shown by pSTAT3/STAT3 ratio and fold change (**g**). **h, i** Representative images of osteogenic induction assays (**h**) and their quantification results (**i**) of hBMSCs. The hBMSCs were first cultured in medium supplemented with FBS or NBIF, with either negative control siRNA (siNC) or *STAT3* siRNA (siSTAT3), for 7 days, followed by osteogenic induction and Alizarin Red staining. Scale bar, 100 μm

Enhanced osteogenic differentiation has been reported to be associated with an increase of BMSC self-renewal capacity.^26,27^ To test whether NBIF regulates BMSC self-renewal, we examined transcription levels of osteo-progenitor-related genes in NBIF-fed hBMSC based on the transcriptome data. The results showed notably increased expression of the leptin receptor (*LEPR*), platelet-derived growth factor receptor (*PDGFR*), paired-related homeobox protein 1 (*PRRX1*), MX dynamin like GTPase 1 (*MX1*), Kit ligand (*KITLG*) in NBIF-fed hBMSCs (Fig. 3a). We confirmed upregulated expression of *LEPR*, a specific marker for osteo-progenitors in bone marrow,^28^ in NBIF-fed hBMSCs by quantitative RT-PCR analysis (Fig. 3b). Additionally, we isolated mouse BMSCs (mBMSCs) for colony-forming unit fibroblast (CFU-F) assays and observed a significantly higher number of alkaline phosphatase-positive (ALP^+^) colonies in the NBIF-fed group compared to the FBS-fed group (Fig. 3c, d). These results indicate that NBIF is able to support the self-renewal of BMSCs.

The GO Biological Process analysis and Gene Set Enrichment Analysis (GSEA) of RNA-seq data revealed significant activation of multiple pathways in NBIF-cultured hBMSCs, including ERK/MAPK, JAK-STAT3, NOTCH, TGF-β, and NF-κB signaling (Figs. 2k and S5d). To identify pathways functionally linked to NBIF-induced osteogenic/homing marker expression (*LEPR, RUNX2, CXCR4*), we employed specific inhibitors, including PD98059 (MEK inhibitor), AG490 (JAK2/STAT3 inhibitor), DAPT (NOTCH inhibitor), and BAY11-7082 (NF-κB inhibitor), to test their effect on NBIF-fed BMSCs. The JAK/STAT3 selective inhibitor AG490 significantly decreased NBIF-induced *LEPR* and *RUNX2* expression (Fig. S6a–d). The JAK-STAT3 pathway has been shown as a crucial signal regulating BMSC osteogenic differentiation and bone development.^29–32^ The Gene Set Enrichment Analysis (GSEA) showed significant enrichment of the JAK-STAT3 signaling pathway in NBIF-fed hBMSCs (Fig. 3e). By Western blotting, we observed an increase of phosphor-STAT3 (Tyr705) in NBIF-fed hBMSCs compared to FBS-fed hBMSC (Fig. 3f, g). Moreover, siRNA-mediated *STAT3* knockdown significantly reduced NBIF-induced expression of *LEPR* and *RUNX2*, and modestly decreased *CXCR4* (Fig. S6e–h). Crucially, *STAT3* knockdown in NBIF-cultured hBMSCs prior to osteogenic induction resulted in significantly impaired mineralization (Alizarin Red S quantification) compared to NBIF + scramble siRNA controls (Fig. 3h, i). These results imply that NBIF may promote BMSC proliferation and osteogenic potential via JAK-STAT3 pathway.

**Fig. 4:**
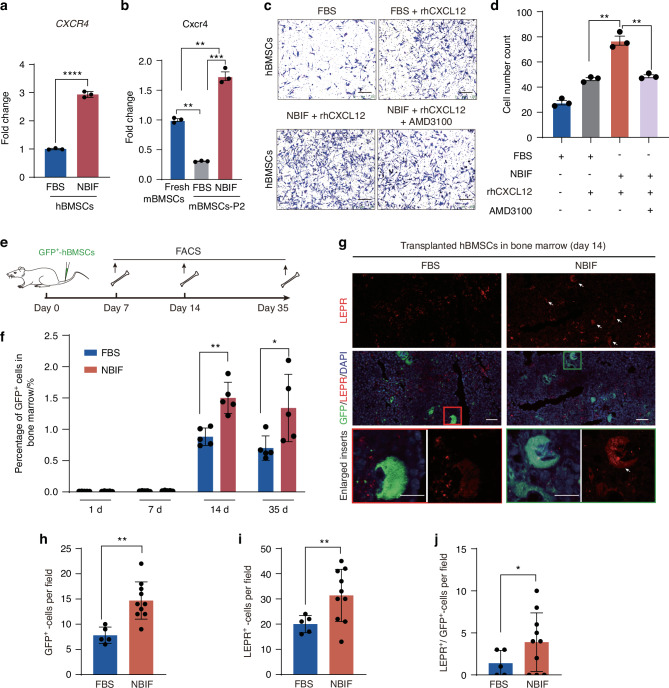
NBIF promotes *CXCR4* expression and enhances hBMSC homing to bone marrow. **a, b** Quantitative RT-PCR analysis of *CXCR4* expression in hBMSCs after 7-day culture (**a**) and in mouse primary mBMSCs after 2 passages (7 days) (**b**). Data [and also in (**d**)] were expressed as mean ± standard error of the mean (SEM) of the fold change across three replicates for each group. *P*-values were obtained from an unpaired *t*-test; **P ≤ 0.01, ****P ≤ 0.000 1. **c, d** Representative images (**c**) and quantification data (**d**) of migratory hBMSCs in the Transwell culture (see the Materials and Methods section for details). rhCXCL12, recombinant human CXCL12 protein; AMD3100, the CXCR4 antagonist. Scale bar, 100 μm. **e** Scheme of the experimental design for mouse transplantation and analysis of GFP-labeled hBMSCs. **f** Quantification of the proportion of GFP^+^-hBMSCs in the whole bone marrow of host mice. Data were expressed as mean ± standard error of the mean (SEM) across indicated replicates for each group. 5 mice for the FBS-fed hBMSCs group and 5 mice for NBIF-fed hBMSCs group at each time point. *P*-values were obtained from an unpaired *t*-test; *P < 0.05, **P < 0.01. **g** Representative immunofluorescent images of markers at 14 days post-transplantation. Scale bar, 10 μm. **h–j** Quantification of GFP^+^- (**h**), LEPR^+^- (**i**) or LEPR^+^; GFP^+^- cells (**j**) in FBS-fed hBMSCs group (*n* = 5 mice) or NBIF-fed hBMSC group (*n* = 10 mice) 14 days post transplantation. Data were expressed as mean ± standard error of the mean (SEM) for each group. *P*-values were obtained from an unpaired *t*-test; *P < 0.05, **P < 0.01

The CXCL12-CXCR4 signaling plays a crucial role in directing the homing of MSCs to sites of injury.^33–35^ We wonder whether NBIF could enhance *CXCR4* expression in BMSCs so as to promote bone marrow homing. RT-PCR analysis detected a significant elevation of *CXCR4* expression level in NBIF-fed hBMSCs (Fig. 4a) and in primary mBMSCs (Fig. 4b) while *Cxcr4* expression in mBMSCs was substantially downregulated after culture in FBS for 2 passages (7 days). Transwell migration assay revealed that NBIF-fed hBMSCs exhibited a significantly enhanced migratory capacity, which was further augmented by stimulation with recombinant human CXCR12. Notably, the administration of the CXCR4 antagonist AMD3100 eliminated the increased migratory ability of NBIF-fed hBMSCs (Fig. 4c, d). These observations suggest that NBIF effectively increases *CXCR4* expression in BMSCs, thereby enhancing their migration and responsiveness to CXCL12. Then, GFP-labeled hBMSCs cultured in either NBIF or FBS medium for 7 days were intravenously injected into the immunocompromised B-NDG mice (NOD.CB17-*Prkdc*^*scid*^*Il2rg*^*tm1*^/Bcgen) to investigate their BM homing ability (Fig. 4e). Flow cytometry analysis revealed that GFP ^+^ -cells were detectable in the bone marrow at 14 and 35 days post-transplantation, with a significantly higher percentage of GFP^+^-cells in the NBIF-fed hBMSCs group compared to the FBS-fed hBMSCs group (Fig. 4f). Immunofluorescent staining demonstrated that more NBIF-fed GFP^+^-hBMSCs or GFP^+^/LEPR^+^-hBMSCs resided in the bone marrow compared to FBS-fed GFP^+^-hBMSCs at 14 days post transplantation (Fig. 4g–j). These observations indicate that NBIF could confer BMSCs greater homing ability to bone marrow likely due to upregulated expression of *CXCR4*.

### NBIF-primed hBMSCs promote bone regeneration

**Fig. 5:**
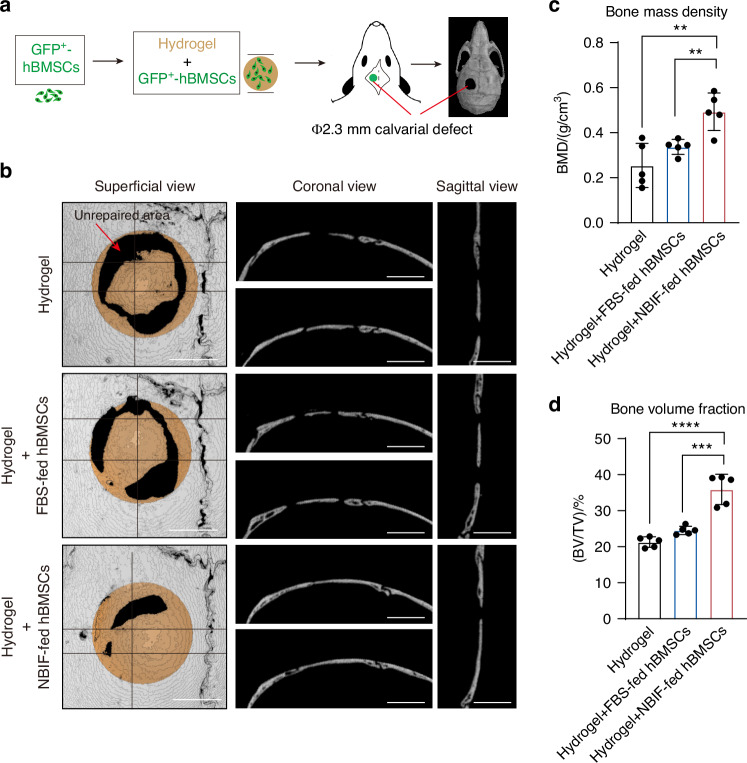
NBIF-primed hBMSCs promote bone regeneration. **a** Scheme of the experimental design for creating mouse calvarial defect and delivering hydrogel or hBMSCs-hydrogel mix. 8-week-old B-NDG mice were used, 5 mice for each repair treatment. Bone regeneration was evaluated by micro-computed tomography (μ-CT) 7 weeks post-treatment. **b** Representative μ-CT images of bone regeneration in the calvarial defect. Horizontal and vertical lines in the left column indicated the dissection lines for coronal (middle column) and sagittal (right column) views, respectively. Typical unrepaired areas were indicated by arrows. Scale bar, 1 mm. **c, d** Bone mass density (BMD) (**c**) and bone volume fraction (BV/TV) (**d**) of regenerated tissues in the defect area at 7 weeks post-surgery. Data were expressed as mean ± standard error of the mean (SEM) across indicated replicates for each group (*n* = 5 mice). *P*-values were obtained from an unpaired *t*-test; **P < 0.01, ***P < 0.001, ****P < 0.000 1

To evaluate the bone regeneration potential of NBIF-fed hBMSCs, we employed a calvarial defect model in 8-week-old B-NDG mice. A 2.3-mm calvarial defect was created, and the defect was filled with hydrogel scaffold alone, or with hydrogel containing GFP-labeled NBIF-fed or FBS-fed hBMSCs (Fig. 5a). Seven weeks post-surgery, micro-computed tomography (Micro-CT) scanning detected regeneration of bone within the defect to different degrees (Fig. 5b). We found that 34% of the defect area was repaired with the NBIF-fed hBMSCs group, while only about 20% of the defect area was repaired with the FBS-fed hBMSCs. Quantification data showed there was a significant increase in bone mass density and bone volume fraction formation for the NBIF-fed hBMSCs group compared to the other two groups (Fig. 5c, d). Overall, these observations suggest that the local delivery of NBIF-cultured hBMSCs significantly enhances the structural integrity and functional regeneration of calvarial bone tissue.

### Proteomic comparison of NBIF, NBS, and ABIF

**Fig. 6:**
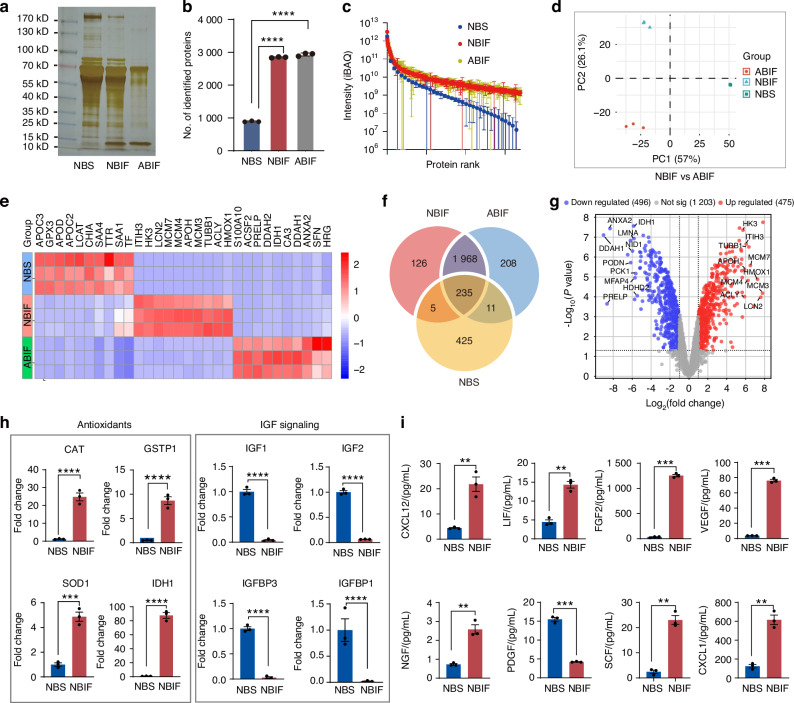
Proteomic profiling of NBIF, NBS and ABIF. **a** General protein patterns after SDS-PAGE gel electrophoresis and staining. An equal amount of protein (20 μg) of each sample was loaded. **b** Number of proteins identified by Mass Spectrometry from the indicated samples. Data were expressed as mean ± standard error of the mean (SEM) across three replicates for each group. *P*-values were obtained from an unpaired *t*-test; ****P < 0.000 1. **c** Dynamics of protein abundances identified in indicated samples. Proteins were quantified using normalized intensity-based absolute quantification (iBAQ) values and log_10_ transformed. **d** Unsupervised principal component analysis (PCA) of protein expression data based on Mass Spectrometry from the indicated samples. **e** Heatmap illustrating the relative expression levels of specific proteins in different samples. **f** The Venn diagram illustrating the number of proteins uniquely identified in each group and the shared proteins among the three groups. Note that each group had three replicates, and only the proteins identified in all three replicates were used for analysis. **g** Volcano plot displaying the measured global differential protein abundance. Volcano plot showing the estimated log_2_ fold changes (log_2_FC) versus the -log_10_ of the *P*-values (y-axis) from Mass Spectrometry. Significant proteins with log_2_FC > 1, *P* < 0.05 were shown in red, and log_2_FC < -1, *P* < 0.05 in blue. **h** Expression levels of antioxidant proteins and IGF related proteins in NBIF compared to NBS from mass spectrometry. Data [and also in (**i**)] were expressed as mean ± standard error of the mean (SEM) across three replicates for each group. *P*-values were obtained from an unpaired *t*-test; **P < 0.01, ***P < 0.001, ****P < 0.000 1. **i** Luminex assay results displaying levels of selected major factors in NBIF and NBS

The above observations suggest that NBIF contains special components empowering BMSCs to have greater osteogenic capacity with enhanced chemotactic homing. Then, we decided to compare the protein composition of NBIF, neonatal bovine serum (NBS), and ABIF. We obtained NBIF and NBS from three newborns (1-7 days old) and ABIF from three 36-month-old cattle. (Fig. 6a), and performed label-free protein quantitative mass spectrometry. This assay identified a total of 2 847 proteins from NBIF, 2 934 proteins from ABIF, and 891 proteins from NBS (Fig. 6b). The protein contents ranked by intensity showed similar distributions between NBIF and ABIF but differed significantly from NBS (Fig. 6c). The Principal Component Analysis (PCA) demonstrated clear separation among NBIF, ABMS and NBS (Fig. 6d), and different sets of proteins were enriched in different groups (Fig. 6e), which indicates their distinct biological functions.

A total of 2 203 proteins were commonly expressed in all NBIF and ABIF samples, among which 1 203 proteins existed with comparable levels in both NBIF and ABIF (Fig. 6f), along with 475 upregulated and 496 downregulated factors in NBIF (Fig. 6g). Comparison of protein components in between NBIF and paired NBS identified 240 co-existing factors, 436 as NBS unique proteins and 2 094 as NBIF unique proteins (Fig. 6f). The Gene Ontology (GO) network analysis indicated that the upregulated proteins in NBIF primarily participated in glutathione metabolic process, which may elevate antioxidant capacity,^36^ ribonucleoside monophosphate metabolic process, which regulates cell proliferation, differentiation, and survival, and positive regulation of alpha-beta T cell activation, which may affect immunomodulatory function of cultured cells^37^ (Fig. S7a). The downregulated proteins in NBIF were associated with fibrinolysis affecting cell adhesion,^38^ IGF signaling pathway regulating cell proliferation,^39^ reverse cholesterol transport that may bias differentiation,^40^ and etc. (Fig. S7b). Interestingly, the top 2 enrichments of biological processes in ABIF involved oxidative and fatty acid metabolism, which may increase cellular oxidative stress, suggesting a potential contribution of those enriched proteins to ABIF-induced cell senescence (Fig. S7c). Notably, the antioxidant enzymes CAT, GSTP1, SOD1, PRDX4, and GPX1 were enriched in NBIF, while insulin signaling-related proteins such as IGF1, IGF2, IGFBP1, and IGFBP3 were downregulated (Fig. 6h). Interestingly, adding recombinant human IGF1 along with NBIF could significantly enhance hBMSCs proliferation compared to NBIF alone (Fig. S7d). Further Luminex analysis revealed significantly elevated levels of several cytokines and growth factors, including LIF, bFGF, VEGF, HGF, NGF, and SCF in NBIF compared to NBS (Fig. 6i). These differences in composition may explain why NBIF- versus FBS-cultured hBMSCs possess distinct features.

### HMGB1, LTF, and NGF in NBIF help maintain the features of BMSCs

We hypothesize that NBIF primes the osteogenic potential of BMSCs by activating specific gene expression via ligand-receptor interactions. To identify bioactive ligands in NBIF (Fig. S8a), we first applied the Human Protein Atlas (HPA) database (https://www.proteinatlas.org/) to screen all known ligands that are present in NBIF, NBS, and ABIF. We identified 99 ligands that were uniquely enriched in NBIF. Then, we evaluated these ligands’ potential ability to regulate gene expression in NBIF-fed hBMSC by calculating ligand activity on these genes (Fig. S8b). Among the 99 ligands, 56 demonstrated significant ligand activity in modulating the gene expression profile of NBIF-induced hBMSCs. We picked up the upregulated genes in NBIF-cultured hBMSCs that have been reported to be involved in osteogenesis,^26–28^ and further analyzed their correlation with identified ligands. The result indicates that these 56 NBIF-derived ligands may regulate BMSC osteogenesis (Fig. S8c).

To further narrow down the number of candidate osteogenic regulatory proteins in NBIF, we employed Size-Exclusion High-Performance Liquid Chromatography (HPLC) to fractionate NBIF based on protein size. We collected five fractions from the flow-through, which were then cold-dried and resuspended in PBS (Fig. S9a). To evaluate impacts on osteogenic potential, each fraction with an equal amount was supplied to the culture medium for culturing hBMSCs, followed by Alizarin Red staining. Results indicated that hBMSCs cultured with the BM3 fraction exhibited an osteogenic capacity comparable to that with the 10% NBIF supplement (Fig. S9b). This observation suggests that the BM3 fraction contains proteins capable of promoting the osteogenic differentiation of BMSCs. SDS-PAGE analysis revealed that proteins within the BM3 fraction had molecular weights predominantly ranging between 25–100 kD (Fig. S9c). Interestingly, this molecular weight range included some of the 56 NIBF-derived ligands, i.e., HMGB1 (25 kD), HGF (83 kD), CAT (60 kD), LTF (78 kD), and NGF (27 kD), suggesting that they likely play a role in priming the osteogenic capacity of BMSCs. Notably, HMGB1 and LTF were abundant in NBIF, as evaluated by mass spectrometry and ELISA (Fig. S10a, b).

**Fig. 7:**
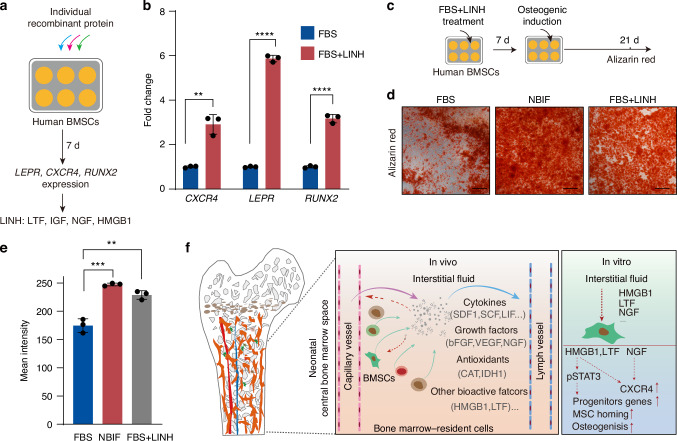
HMGB1, LTF, and NGF in NBIF help preserve features of hBMSCs. **a** Strategy to test effect of selected proteins on hBMSCs. **b** The effect of LINH cocktail supplemented factors on the *CXCR4*, *LEPR* and *RUNX2* expression in cultured hBMSCs. Cells were cultured for 7 days in FBS-supplied standard culture medium with addition of indicated specific factors, followed by qRT-PCR analysis. Data [and also in (**e**)] were expressed as mean ± standard error of the mean (SEM) across three replicates for each group. *P*-values were obtained from an unpaired *t*-test; **P < 0.01, ****P < 0.000 1. **c** Strategy to test the effect of LINH cocktail on hBMSCs osteogenic ability. The hBMSCs were cultured in medium supplemented with FBS, NBIF, or LINH cocktails for 7 days, followed by osteogenic induction and Alizarin Red staining. **d** Representative images of osteogenic induction assays. **e** Quantification results of Alizarin Red staining of indicated group. Scale bar, 100 μm. **f** Summary of the study. The interstitial fluid in the bone marrow of neonatal animals contains various soluble proteins secreted by different bone marrow resident cells, including cytokines, growth factors, antioxidant proteins, and other bioactive proteins, which help maintain local MSCs. When applied to in vitro culturing hBMSCs, the NBIF factors promote the expression of LEPR^+^ hBMSC progenitor genes and the chemokine receptor *CXCR4*, thereby enhancing osteogenic capacity and the homing-to-BM ability

Next, we tested the effects of recombinant human HMGB1, HGF, CAT, LTF, and NGF, along with IGF1, on the expression of hBMSC osteogenic differentiation and chemotaxis markers at a concentration of 100 ng/mL (Fig. 7a). Individual factor was added to the FBS-containing medium for culturing hBMSCs for 7 days. The results indicated that LTF, NGF, and HGF each significantly upregulated *CXCR4* expression; HMGB1 and NGF effectively increased the expression of the hBMSC precursor marker *LEPR*; while NGF and IGF mildly enhanced *RUNX2* expression (Fig. S10c). Then, we tested individual factors across a range of concentrations for their ability to induce *LEPR*, *RUNX2*, and *CXCR4* expression in FBS-cultured hBMSCs after 7 days. Notably, the results showed that HMGB1 at 1 000 ng/mL, IGF1 at 50 ng/mL, LTF at 1 000 ng/mL and NGF at 500 ng/mL significantly upregulated the expression of these three marker genes (Fig. S11a–c). Finally, we evaluated the combined effect of LTF, IGF, NGF, and HMGB1, referred to as “LINH cocktail”, with the optimized dosage for treatment. The results demonstrated that the LINH cocktail significantly promoted the expression of *LEPR*, *RUNX2*, and *CXCR4* in hBMSCs (Fig. 7b). Moreover, hBMSCs pre-cultured for 7 days in FBS + LINH cocktail exhibited enhanced osteogenic differentiation (Fig. 7c–e). Thus, the LINH cocktail may mimic NBIF, at least partially, to enhance the osteogenic and bone marrow-homing potential of hBMSCs.

## Discussion

In this study, we have demonstrated that neonatal bone marrow interstitial fluid, which constitutes bone marrow microenvironment and regulates the fate of bone marrow progenitor cells, could replace fetal bovine serum to support the expansion of hBMSCs in vitro (Fig. 7f). Importantly, NBIF not only supports hBMSCs growth in vitro but also enhances their osteogenic differentiation potential with the expansion of LEPR^+^ BMSC progenitors and promotes bone defect repair in a calvarial defect mouse model compared to FBS. The gain of these improvements was supported by transcriptome shifts towards multiple organ developmental and repair processes, such as ossification, wound healing, and bone formation.

It has been reported that freshly isolated BMSCs can effectively home to bone after systemic delivery into syngeneic mice; however, BMSCs upon in vitro culturing exhibit greatly reduced homing potential to bone due to rapid loss of Cxcr4 expression.^20,41^ We show that, compared to standard FBS-supplemented culture, the addition of NBIF significantly enhances the expression of the chemokine receptor CXCR4 in primary hBMSCs. The sustained expression of CXCR4 upon NBIF pretreatment could enhance the BMSCs response to the chemoattractant CXCL12, abundant in the bone marrow, thereby promoting BMSCs homing to bone.

Bovine serum (FBS/NBS) has been widely used and shown to support the in vitro expansion of a wide range of human cell types effectively, suggesting that many key factors are conserved and functionally cross-reactive across species. Our experimental data support the idea that bovine NBIF likely contains biologically active factors capable of interacting with human cells, although species-specific differences cannot be entirely excluded. Through bioinformatics analysis, size-based HPLC fractionation and biological functional assays, we identified HMGB1, LTF and NGF, which are significantly enriched in NBIF, as ligands to promote hBMSC osteogenic differentiation and *CXCR4* expression. HMGB1/2, typically recognized as a damage-associated molecular pattern molecule released from the nucleus, has been reported to play a critical role in promoting bone and heart regeneration following injury.^42–47^ Several HMGB1 receptors have been identified, including Toll-Like Receptor 2/4 (TLR2/4), Advanced Glycosylation End-Product Specific Receptor (AGER) and CXCR4.^48^ Intriguingly, *CXCR4* and *TLR4* expression were increased in the NBIF-primed hBMSCs (Figs. 4a, b, and S12b) and could be potential receptors for HMGB1. Our data show that JAK-STAT3 signaling is significantly activated in NBIF-primed hBMSCs (Fig. 3e, f), and critical for *LEPR* expression during hBMSC expansion in vitro (Fig. S6a, f). It has been reported that HMGB1 drives microglial activation via STAT3/p65 axis.^49^ Whether HMGB1 promotes BMSC self-renewal through JAK-STAT3 signaling needs further study. LTF has also been documented to promote bone development, which is primarily secreted by neutrophils in the bone marrow.^50–53^ Nerve growth factor (NGF), a critical factor for neurogenesis and development,^54^ is more abundant in NBIF than in NBS (Fig. 6i). Moreover, the neurodevelopmental signaling pathway is the most significantly activated signaling pathway in the NBIF-primed hBMSCs (Fig. 2k). Interestingly, NGF has been shown to enhance calvarial defect repair in vivo by recruiting and supporting osteogenic precursor cells.^55,56^ Our results demonstrated that NGF significantly increases *CXCR4* expression (Fig. S11a). Importantly, the application of the human homolog-based LINH cocktail consisting of recombinant human proteins NGF, IGF, LTF, and HMGB1 can partially mimic the osteogenic differentiation-promoting effect of NBIF on hBMSCs while enhancing *CXCR4* expression.

Despite the promising findings, our study still has some limitations. We primarily characterized the protein components of NBIF and validated its biological functions through in vitro assays. The effect of the LINH cocktail on cultured hBMSCs properties should be investigated more extensively, and the application of the LINH-fed hBMSCs to bone repair should be explored in the future. Additionally, while our focus was on the protein composition of NBIF, it is important to investigate other components, such as secreted metabolic molecules and their effects on progenitor cells within the bone marrow.

In summary, our data provide the first detailed report demonstrating that neonatal bone marrow interstitial fluid contains bioactive secreted proteins, including a variety of growth factors, cytokines, and other components. The interstitial fluid supports the expansion of hBMSCs and promotes their osteogenic potential, suggesting that the addition of NBIF may provide in vitro culture conditions more similar to the in vivo bone marrow microenvironment. The addition of specific bone marrow niche factors in the FBS culture medium largely mimics the osteogenic differentiation-promoting and bone marrow homing effects of NBIF on hBMSCs, which may be used for clinical bone regeneration.

## Materials and methods

### Isolation of bone marrow interstitial fluid

Bone marrow tissue was harvested from the long bones of the leg from healthy, culled newborn (male, 1–7 days) and adult (male, 36 months) Holstein cattle, which were raised in Longmen County Hefeng Farm, Guangdong Province, China. Interstitial fluid was prepared following previously published protocols with modifications.^6,22^ Briefly, bone marrow was harvested from long bones and immediately mixed with two times volume of ice-cold PBS containing a protease inhibitor cocktail (Roche) and heparin. The mixture was incubated on ice for 30 minutes and then centrifuged at 2 000 *g* for 10 minutes at 4 °C. The supernatant was collected and centrifuged again at 10 000 *g* for 30 minutes at 4 °C to remove cell debris. The supernatant was filtered through a 0.22-μm membrane (Thermo Fisher Scientific, USA), followed by filtration 3 times through 0.1-μm membranes (Thermo Fisher Scientific) to ensure removal of mycoplasma. The protein concentration was quantified using the BCA Protein Assay Kit (Beyotime, China). To minimize variability, bone marrow interstitial fluid (NBIF) from five animals was pooled for use in this study. The final protein concentrations of NBIF, ABIF, and NBS were all adjusted to around 0.25 mg/mL, and the samples were stored at -80 °C for subsequent experiments.

### Source and culture of MSCs

Human primary bone marrow-derived MSCs (iCell) and adipose-derived MSCs (iCell) were purchased from iCellBioscience (Shanghai, China). Human umbilical cord-derived MSCs were isolated via an adherent method.^57^ The umbilical cord biological samples used in this study were collected in accordance with the approval of the Sun Yat-sen Memorial Hospital’s ethics committee. The umbilical cord was briefly rinsed with 70% ethanol and washed 3 times with cold PBS, and the blood vessels within the umbilical cord were carefully removed. The umbilical cord was then cut and dissected into small fragments approximately 2 mm in diameter and washed 3 times with cold PBS. Subsequently, the small fragments were plated onto 10 cm culture dishes, allowing them to adhere for 10 minutes. Fresh medium containing 10% fetal bovine serum (Gibco, USA) and 1% penicillin/streptomycin (Thermo Fisher Scientific) was added to the culture dishes. The cultures were maintained at 37 °C with 5% CO_2_, and the medium was changed every 3 days. After 10 days, the remaining umbilical cord tissues were removed, and the adhered cells were considered as passage 0. Cells were passaged at approximately 80% confluence. Passages 3-4 of the umbilical cord-derived MSCs (UCMSCs) were used for in vitro experiments. Mouse primary BMSCs were isolated as previously reported.^58^ Mouse (1–2 months old) tibia and femur were cut into pieces and digested with Type I collagenase (Gbico) to obtain a single-cell suspension. The single-cell suspension after treatment with the red blood cell lysate (Solarbio, China) was seeded in a 10 cm dish and was cultured with DMEM/F12 (Gibco) containing 10% fetal bovine serum(Gibco), 2% Penicillin-Streptomycin (Thermo Fisher Scientific). Cells were maintained at 37 °C with 5% CO_2_ and passaged at approximately 80% confluence. The CD45-CD11b-Sca-1^+^CD29^+^ cells were confirmed as bone marrow stromal cells(BMSCs) by flow cytometry.^59^ Passages 1-3 of the BMSCs were used for in vitro experiments. For subsequent studies, commercially available FBS (Gibco) or homemade NBIF was added to the DMEM/F-12 basal medium with 1% penicillin/streptomycin. For inhibitor treatment, 50 μmol/L PD98059 (MEK inhibitor), AG490 50 μmol/L (JAK2/STAT3 inhibitor), 5 μmol/L DAPT (NOTCH inhibitor), and 5 μmol/L BAY11-7082 (NF-κB inhibitor) were applied to BMSCs medium together with NBIF for 7days. To compare the effects of NBIF, ABIF, and FBS on cell growth, we added them with the same protein amount to the DMEM/F-12 basal medium. Normally, NBIF, ABIF, or FBS stock was added with 10% v/v to reach a final concentration at 25 μg/mL. For recombinant protein treatment, recombinant human HMGB1, HGF, CAT, LTF, NGF, and IGF1 with indicated concentrations, which were purchased from Sino Biological or Tsingke Biotechnology Co., Ltd., were individually or in combination added to the 10% FBS-containing medium for culturing hBMSCs for 7 days.

### siRNA transfection

For STAT3 knockdown, three siRNA oligonucleotides targeting human STAT3 (siRNA1-sense: 5’-CACAAUCUACGAAGAAUCA(dT)(dT)-3’; siRNA2-sense: 5’- GUCAUUAGCAGAAUCUCAA(dT)(dT)-3’; siRNA3-sense: 5’- CCAACAAUCCCAAGAAUGU(dT)(dT)-3’) and non-targeting control siRNA (siNC-sense: 5’-GUAUGACAACAGCCUCAAG(dT)(dT)-3’) were synthesized by Tsingke Biotechnology. Cells were plated at approximately 80% confluence at the time of transfection. Lipofectamine RNAiMAX transfection reagent (Invitrogen) was applied for siRNA transfection. RNA-lipid complexes were prepared according to the manufacturer’s protocol and subsequently added to the cells, and we mixed the three siRNA for knockdown experiments. The cells were incubated at 37 °C for 72 hours to allow for effective gene silencing. Following incubation, cells were harvested to assess knockdown efficiency via qPCR and Western blot analyses, or they were used directly for subsequent functional assays. The efficiency of STAT3 silencing was confirmed prior to further experimental procedures. The primers pairs for qPCR are as follow: *hSTAT3*-F: 5’- CAGCAGCTTGACACACGGTA -3’, *hSTAT3*-R: 5’- AAACACCAAAGTGGCATGTGA -3’; *hCXCR4*-F: 5’- CTCCTCTTTGTCATCACGCTTCC -3’, *hCXCR4*-R: 5’- GGATGAGGACACTGCTGTAGAG -3’; *hLEPR*-F: 5’- CTTGTGTGCAGTCTATGCTGT -3’, *hLEPR*-R: 5’- CCATGACAACTGTGTAGGCTG-3’; *hCDNK1A*-F: 5’- TGTCCGTCAGAACCCATGC-3’, *hCDNK1A*-R: 5’- AAAGTCGAAGTTCCATCGCTC-3’; *hCDNK2A*-F: 5’- GATCCAGGTGGGTAGAAGGTC-3’, hCDNK2A-R: 5’- CCCCTGCAAACTTCGTCCT -3’; *hGAPDH*-F: 5’- GTCTCCTCTGACTTCAACAGCG -3’, *hGAPDH*-R: 5’- ACCACCCTGTTGCTGTAGCCAA -3’.

### Flow cytometry analysis of expanded hBMSCs

For flow cytometry analysis, 1 × 10^6^ dissociated hBMSCs were suspended in 100 μL of 2% FBS in PBS and incubated with primary antibodies anti-human CD45-BV510 (1:100, 563204, BioLegend); anti-human CD34-PE/Cy7 (1:100, 343616, BioLegend); anti-human CD90-APC (1:100, 328114, BioLegend); anti-human CD105-PE (1:100, 560839, BD Pharmingen); and anti-human CD73-FITC (1:100, 561254, BD Pharmingen); anti-mouse Cd45-BV510 (1:100, Biolegend, 103138); anti-mouse CD11b-APC(1:100, Biolegend, 101212); anti-mouse Ly-6A/E (Sca-1)- PE/Cyanine7 (1:100, Biolegend, 122514); anti-mouse CD29-FITC (1:100, Biolegend, 102206); DAPI (1:200, Biolegend, 422801) for 30 minutes on ice. Fluorescence was detected using a BD FACSAria III (BD Biosciences) or CytoFLEX S(Beckman) flow cytometer, and data were analyzed using FlowJo software.

### Senescence-associated β-galactosidase staining

The senescence of hBMSCs treated with FBS, NBIF, or ABIF was assessed using a Senescence Cells Histochemical Staining Kit (Beyotime) according to the manufacturer’s protocol. Images were taken from random areas of each well, and the percentage of SA-β-gal^+^ cells was calculated. Cells with intracellular blue deposits were considered as senescent cells.

### Karyotyping of hBMSCs

The karyotyping of hBMSCs was performed by Guangzhou Zhetai Biotechnology Co., Ltd. Briefly, cells at approximately 80% confluence were treated with 200 ng/mL colcemid for 2 h at 37 °C to arrest cell division. Subsequently, cells were collected and centrifuged at 1 200 r/min for 5 minutes. The cell pellet was washed with PBS for 2 times. The cells were then incubated in pre-warmed hypotonic KCl solution at 37 °C for 20–30 min, and fixed in methanol:acetic acid (3:1, v/v) for 3 min, centrifuged, and removal of the supernatant. The cell pellet was gently resuspended in fresh methanol:acetic acid (3:1, v/v), and slides were prepared by dropping the cell suspension onto pre-cooled slides, then air-dried at 75 °C for 3 h. The slides were then stained with Giemsa solution after enzymatic digestion with trypsin and washing with PBS. Chromosomal images in metaphase were captured for karyotype analysis using the Ikaros karyotyping system.

### Multiplex Luminex and ELISA assay

A panel of cytokines, chemokines, and growth factors was analyzed in bovine-derived samples (NBIF, ABIF and NBS) and hBMSCs-derived samples (supernatant from pretreated hBMSCs) utilizing the Luminex xMAP-based multiplex assay (Millipore) according to the manufacturer’s protocol, including: NGF, CCL2 (MCP-1), CCL3 (MIP-1α), CCL4 (MIP-1β), CCL7 (MCP-3), CCL11 (Eotaxin), CTACK (CCL27), CXCL1 (GRO-α), CXCL9 (MIG), CXCL10 (IP-10), CXCL12 (SDF-1α), FGF2, G-CSF, GM-CSF, HGF, IFN-α2, IFN-γ, IL-1α, IL-1β, IL-1ra, IL-2, IL-2Rα, IL-3, IL-4, IL-5, IL-6, IL-7, IL-8, IL-9, IL-10, IL-12 (p40), IL-12 (p70), IL-13, IL-15, IL-16, IL-17, IL-18, LIF, M-CSF, MIF, PDGF, CCL5(RANTES), SCF, SCGF-β, TNF-α, TNF-β, TRAIL, and VEGF. Triplicated samples were analyzed for each group. The NBS/NBIF HMGB1 and LTF were quantified by Bovine HMGB1 ELISA Kit (Abebio, AE39883BO) and Bovine LTF ELISA Kit (Abebio, AE34833BO), respectively, according to the manufacturer’s instructions.

### Migration assay

A Transwell chamber (Corning) with 8 μm diameter membrane filter was used for migration assay according to the manufacturer’s instructions. hBMSCs pretreated with NBIF or FBS were seeded in the upper chamber at a density of 5 × 10^5^ cells/well in serum-free medium. The lower chamber was filled with the same medium supplemented with 10% FBS. To assess the effect of CXCL12, recombinant human CXCL12 was added to the lower chamber at a concentration of 100 ng/mL, with or without 50 nmol/L CXCR4 inhibitor AMD3100 (Medchemexpress) for 24 hours. Migrated cells were stained with crystal violet staining solution, and were counted in 3 randomly chosen visual fields (200×) under a microscope (Zeiss).

### Colony-forming unit-fibroblast (CFU-F) assay

For CFU-F assays, expanded hBMSCs or fresh isolated mBMSCs were seeded in 6-well plate at a density of 1 000 cells/well in basal medium (DMEM/F-12 supplemented with 1% Penicillin/Streptomycin), supplied with NBIF, ABIF or FBS, respectively. Cells were incubated at 37 °C with 5% CO_2_, with half of the medium changed every other day. At day 10, cells were fixed and stained with the Alkaline Phosphatase (ALP) staining solution (Beyotime). Adherent ALP-positive colonies were quantified.

### Multilineage differentiation assays

For osteogenic differentiation, hBMSCs at 100% confluence were plated in MesenCult™ Osteogenic differentiation medium (STEMCELL Technologies). The medium was changed every 3 days over periods of 3 weeks according to the manufacturer’s instructions. For adipogenic differentiation, hBMSCs at 100% confluence were cultured in MesenCult™ adipogenesis differentiation medium (STEMCELL Technologies). The medium was changed every 2 days for 3 weeks according to the manufacturer’s instructions.

For chondrogenic differentiation, 5 × 10^5^ of hBMSCs were added to chondrogenic medium (DMEM/F-12, 1% Insulin-Transferrin-Selenium (ITS) (Gibco), 100 nmol/L dexamethasone (Sigma), 1% Penicillin-Streptomycin, 10 ng/mL TGF-β (Novoprotein), 50 μg/mL L-ascorbic acid 2-phosphate, 40 μg/mL L-proline, and 100 μg/mL sodium pyruvate) in 15 mL tube and centrifuged for 5 minutes at 300 × *g* to form a pellet. Cell pellets were incubated at 37 °C with 5% CO_2_, and the medium was changed every 3 days for 4 weeks.

### Oil Red O staining

The lipid drop was stained by Oil Red O Staining Kit (Beyotime), according to the manufacturer’s instructions. hBMSCs were washed with phosphate-buffered saline (PBS) and fixed with 4% paraformaldehyde for 30 minutes at room temperature. After fixation, cells were rinsed again with PBS and stained with Oil Red O working solution for 30 minutes. The stained cells were then washed 4–5 times with PBS and observed under a light microscope for qualitative evaluation of lipid accumulation.

### Alkaline Phosphatase (ALP) activity assay

Alkaline phosphatase (ALP) activity was detected by Alkaline Phosphatase Color Development Kit (Beyotime), according to the manufacturer’s instructions. BMSCs were pretreated under the indicated conditions, and the culture medium was replaced with the experimental medium. Cells were briefly washed with PBS, BCIP/NBT staining solution was applied to ensure coverage. After 5–30 minutes’ incubation, BCIP/NBT was removed and washed twice with distilled water.

### Alizarin red staining

To evaluate mineralization, hBMSCs were washed with cold PBS and fixed with 70% ethanol for 15 minutes on ice. Following fixation, cells were washed with distilled water and stained with 2% Alizarin red solution (Beyotime) for 5 minutes. After staining, cells were washed with distilled water and images were taken under a microscope to assess mineral deposition.

### Alcian blue staining

After removing the culture medium, the cell pellet was washed with PBS and fixed with 4% PFA for 30 minutes. The pellet was stained with 1% Alcian Blue solution (Sigma) in 3% acetic acid (pH 2.5) for 30 minutes, and washed three times with 0.1 N HCl followed by rinsing with PBS to remove excess dye. The stained pellets were then subjected to paraffin embedding and sectioning for microscopy.

### Animals and surgical procedures

Mouse studies were conducted in accordance with protocols approved by the Institutional Animal Care and Use Committee of Guangzhou National Laboratory. For hBMSCs homing experiments, 8 to 10-week-old female B-NDG (NOD.CB17-*Prkdc*^*scid*^*Il2rg*^*tm1*^/Bcgen) mice (Biocytogen) were utilized for this study. GFP-labeled hBMSCs were cultured in either NBIF-supplied medium or standard FBS medium for 7 days. The expanded cells were then systematically delivered into mice via intravenous injection. Bone marrow cells were harvested at 0 day, 7 days, 14 days, and 35 days post-transplantation to quantify GFP^+^-cells using flow cytometry and immunostaining. Mice hind limb at 14-day post-transplantation were harvested for immunofluorescent staining. For the calvarial defect model, a cranial defect (2.3 mm diameter) was created at the left side region of the calvarium using a dental trephine. The mice were randomly divided into three experimental groups: (1) Scaffold Only group (hydrogel scaffold); (2) FBS-hBMSCs Scaffold group (hydrogel+ FBS-hBMSCs); (3) NBIF-hBMSCs Scaffold group (hydrogel+ NBIF-hBMSCs). To prepare the scaffold/hBMSCs mix, TrueGel3D™ hydrogel (Millipore Sigma) was made according to the manufacturer’s instructions on ice, 5×10^5^ cells were mixed with the hydrogel and allowed to solidify at 37 °C, forming pellets with a diameter of approximately 3 mm. The calvarial defect was filled with indicated Scaffold group, and skin was closed with surgical suture, The mice received daily subcutaneous injections of 5 mg/kg meloxicam for pain relief during the first week after surgery. All surgical procedures were conducted under isoflurane anesthesia (3%). Mice were sacrificed at 7 weeks post-surgery, and calvarial specimens were harvested for further assessments.

### Micro-CT analysis

Following the surgical creation of calvarial defects, mice (*n* = 5 per group) were euthanized at 7 weeks post-surgery. Calvarial specimens were harvested and fixed overnight in 4% paraformaldehyde (PFA) at 4 °C. The fixed samples underwent micro-CT scanning at a resolution of 4 µm. Three-dimensional (3D) reconstructions of the calvarium were performed and analyzed using IMALYTICS Preclinical 2.1 software. A bone defect area of 2.3 mm (referred to 0-day post-surgery) was defined as a region of interest (ROI) to evaluate both bone volume and tissue volume for the calculation of bone volume/tissue volume (BV/TV) and bone mineral density (BMD).

### Immunofluorescent staining

Hind limb femur from mice were fixed in 4% paraformaldehyde, decalcified, and then embedded in paraffin for sectioning. The sections were deparaffinized, and antigen retrieval was performed by microwave-heating the slides in 10 mmol/L citrate buffer (pH 6.0). Samples were incubated with a blocking solution containing 5% bovine serum albumin and 0.03% Triton X-100 in PBS to reduce nonspecific binding. Primary antibodies were applied at 4 °C overnight, including anti-GFP (1:100 dilutions, AF2724, Santa Cruz), and anti-LEPR (1:200 dilutions, NBP2-67296, Abcam). After washing, sections were incubated with appropriate Alexa Fluor secondary antibodies (Goat anti-mouse Alexa Fluor 488, A11001; Goat anti-rabbit Alexa Fluor 647, A32733, Thermo Fisher Scientific) at a dilution of 1:500 for 2 hours at room temperature. Nuclei were stained with 0.1 µg/mL DAPI (Beyotime). Stained samples were examined by Carl ZEISS LSM900 confocal microscope.

### Western blot

For Western blot, cells were lysed on ice using RIPA buffer (Beyotime, P0013B). The proteins were separated by electrophoresis on a 4%-20% precast gel (Tanon) and electrotransferred to a PVDF membrane (Millipore). The membrane was blocked with 5% BSA at room temperature for 1 hour. The membrane was incubated overnight at 4 °C with the following primary antibodies: GAPDH (1:2 000, 60004-1-IG, Proteintech), anti-pSTAT3-Ser705 (1:1 000, 9145, CST), and anti-STAT3 (1:1 000, 9139, CST). The membrane was incubated with secondary antibody (1:3 000, Dylight 800, Goat Anti-Rabbit IgG, A23910, Abbkine; 1:3 000, Dylight 680, Goat Anti-Mouse IgG, A23720, Abbkine) for 1 hour at room temperature. Fluorescent signals were detected using the Odyssey® CLx Infrared Imaging System (Li-COR).

### Bulk RNA sequencing and data analysis

RNA was isolated from cells expanded under the indicated conditions using the RNAsimple Total RNA Kit (Tiangen). RNA quality control was performed using a Nanodrop One (Thermo Fisher Scientific). RNA library preparation was performed by BGI Genomics. Libraries were sequenced on an BGISEQ-500 platform. STAR 2.4.2a was used to align the RNA-seq reads to the reference human genome (hg38). Differential expression analysis was performed using the DESeq2 package (v.1.10.0). Gene Ontology (GO) analysis was performed using the clusterProfiler R package (v3.0.0). Gene set enrichment analysis was performed using GSEA (v.3.0), incorporating gene sets from MSigDB (v.5.0). Normalized enrichment scores (NES) and FDR/q values were used to explore enriched pathways in each phenotype. Data visualization and generating plots were performed in the R environment (v4.3.1) (https://www.r-project.org/), or on SRplot (https://bioinformatics.com.cn/).

### Label-Free quantitative mass spectrometry-based proteomic profiling

For quantitative proteomic profiling, high-abundance proteins were removed from samples by ProteoMiner protein enrichment kit (Bio-RAD). Label-Free quantitative mass spectrometry was performed by BGI Shenzhen Co., Ltd. Triplicated samples were analyzed for each group. Differential expression analysis, along with principal component analysis (PCA) and volcano plots, was performed using the limma R package. Kyoto Encyclopedia of Genes and Genomes (KEGG) pathway analysis and Gene Ontology (GO) analysis were performed using the clusterProfiler R package (v3.0.0). Data visualization and generating plots were performed in the R environment (v4.3.1) (https://www.r-project.org/) or on SRplot (https://bioinformatics.com.cn/). GO terms with corrected *P* values < 0.05 were considered as significantly enriched. GO networks were further re-analysis by Cytoscape v3.10.1 plugins ClueGO. Ligand-activity analysis was performed using NicheNet v2.2.0 (https://github.com/saeyslab/nichenetr).

### High-performance liquid chromatography

NBIF samples were cold-dried and re-suspend in PBS. Samples were then processed through centrifugation to remove insoluble components and then loaded onto a size exclusion chromatography column (Cytiva, 10-600 kD). The flow rate was set at 0.5 mL/min, and detection was conducted at 280 nm to analyze retention times and peak areas of the separated proteins. NBIF fractions were collected according to the peak areas.

### Statistical analysis

Data are presented as mean ± standard error of the mean (SEM) as indicated in the figure legends. Statistical significance between the two groups was assessed using an unpaired *t*-test. Statistical analyses were performed with GraphPad software, and *P* < 0.05 was considered statistically significant.

## Supplementary information

Supplementary Information

Supplementary Table S1

## Data Availability

Bulk RNA-seq data that support the findings of this study have been deposited in the Gene Expression Omnibus under accession codes GSE290284. All other data supporting the findings of this study are available from the corresponding authors on reasonable request.
