# Supplementary material for: Neonatal bone marrow interstitial fluid supports expansion and osteogenic ability of human bone marrow mesenchymal stromal cells
Source: Bone Res. 2025 Dec 15;13:102. doi: 10.1038/s41413-025-00496-z (PMC12703000; doi:10.1038/s41413-025-00496-z)
Supplement: Supplementary file 1 — Supplementary Information [file 41413_2025_496_MOESM1_ESM.docx]

**Supplementary Information**

**Fig. S1**

**
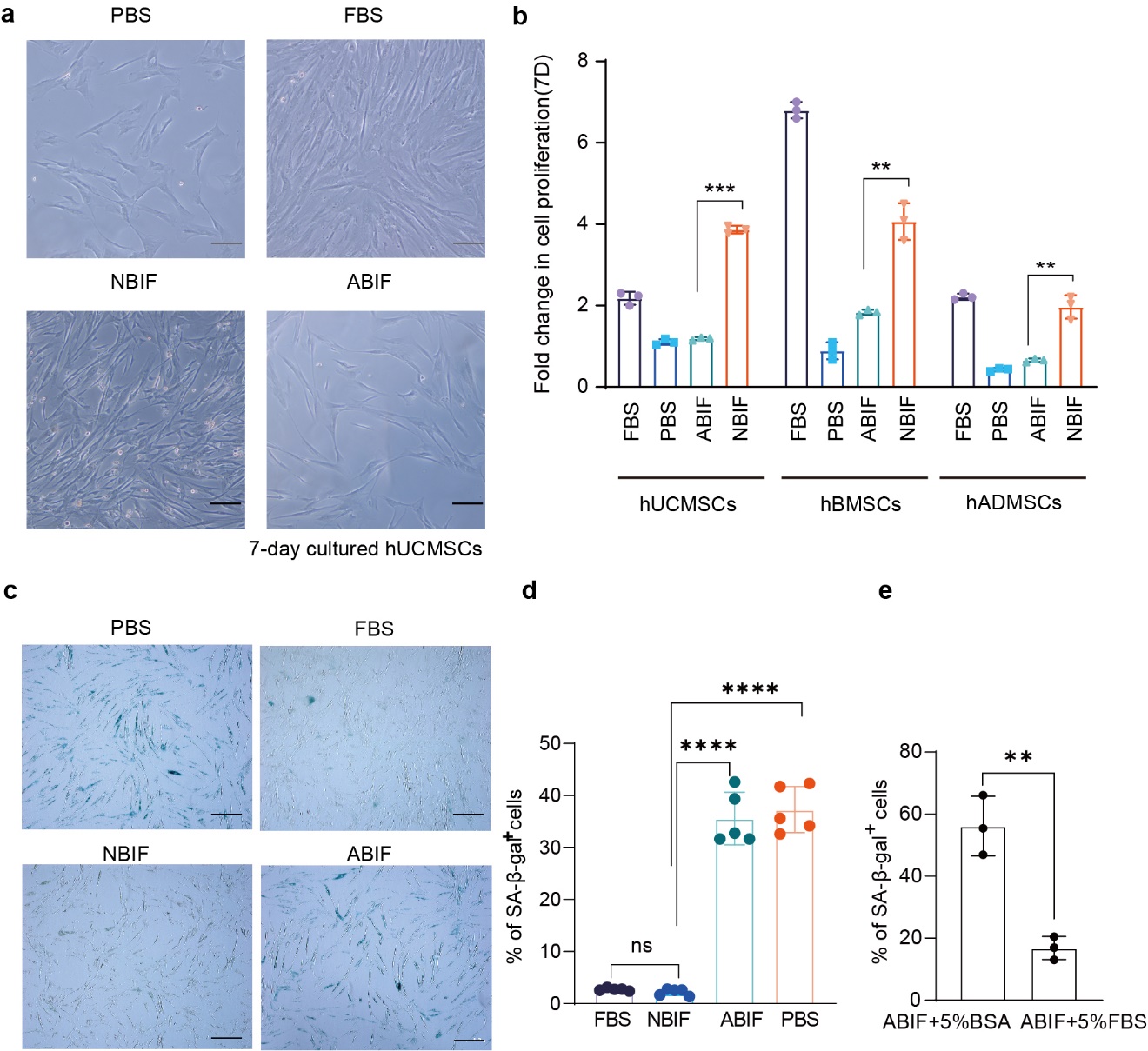
**

**Fig. S1** NBIF but not ABIF supports various human MSCs growth in vitro**. a** Representative morphology of human umbilical cord MSCs (hUCMSCs) cultured under indicated conditions for 7 days. Scale bar, 100 μm. **b** Cell proliferation fold change after 7-day culture under indicated conditions. hADMSCs, human adipose tissue-derived MSCs. Data were expressed as mean ± standard error of the mean (SEM) across three replicates for each group. P-values were obtained from an unpaired *t*-test between two indicated group; ** p<0.01, *** p<0.001. **c** Representative image of SA-β-gal staining for hBMSCs cultured with 10% of indicated agents after 7 days. Scale bar, 100 μm. **d, e** Quantification of SA-β-gal^+^ cells in indicated groups. Data were expressed as mean ± standard error of the mean (SEM) across indicated replicates for each group. P-values were obtained from an unpaired *t*-test; ns, not significant, ** p<0.01, **** p<0.0001.

**Fig. S2**

**
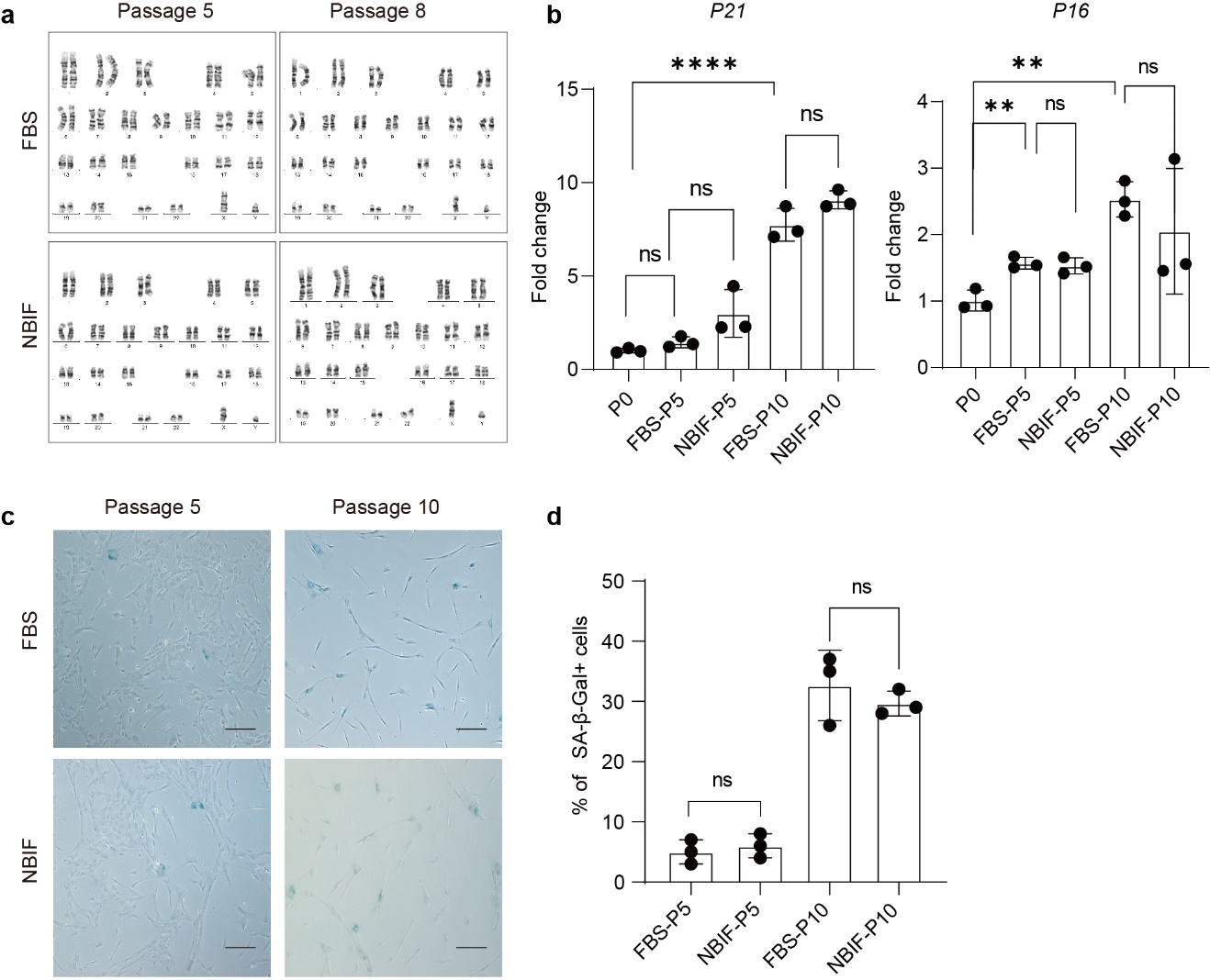
**

**Fig. S2** Karyotyping and detection of senescence-related markers in long-term cultured hBMSCs. **a** Representative karyotypes of cells cultured in medium supplemented with FBS, NBIF at passage 5 and passage 8. **b** Expression levels of *P21* and *P16* in hBMSCs at indicated passages and treatment, evaluated by quantitative RT-PCR. Data (and also in (d)) were expressed as mean ± standard error of the mean (SEM) of the fold change across three replicates for each group. P-values were obtained from an unpaired *t*-test; ns, not significant, ** p ≤ 0.01, *** p ≤ 0.001. **c** Representative image of SA-β-gal staining for hBMSCs cultured with 10% of indicated agents at passage 5 and passage 10. Scale bar, 100 μm. **d** Quantification of SA-β-gal^+^ cells in indicated group in (**c**).

**Fig. S3**

**
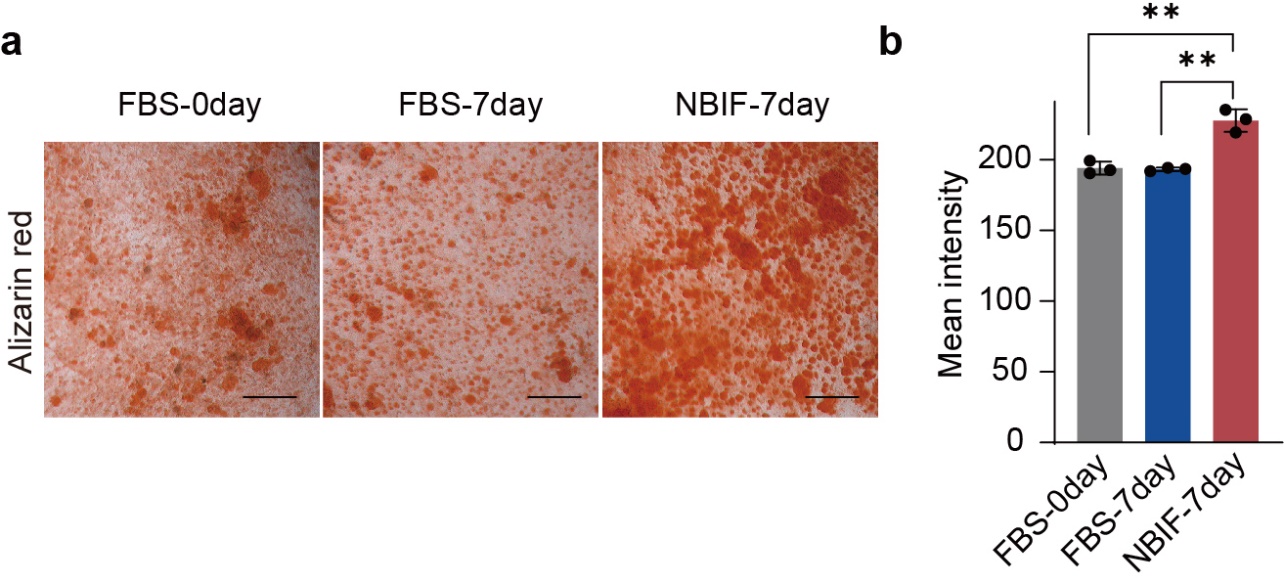
**

**Fig. S3** NBIF enhances hBMSCs osteogenic potential. **a, b** Representative images of osteogenic induction assays (**a**) and their quantification results (**b**) of hBMSCs. The hBMSCs were cultured in medium supplemented with FBS, NBIF for 0 day or 7 days, followed by osteogenic induction and Alizarin Red staining. Data were expressed as mean ± standard error of the mean (SEM) of the fold change across three replicates for each group. P-values were obtained from an unpaired *t*-test; ** p ≤ 0.01. Scale bar, 100 μm.

**Fig. S4**


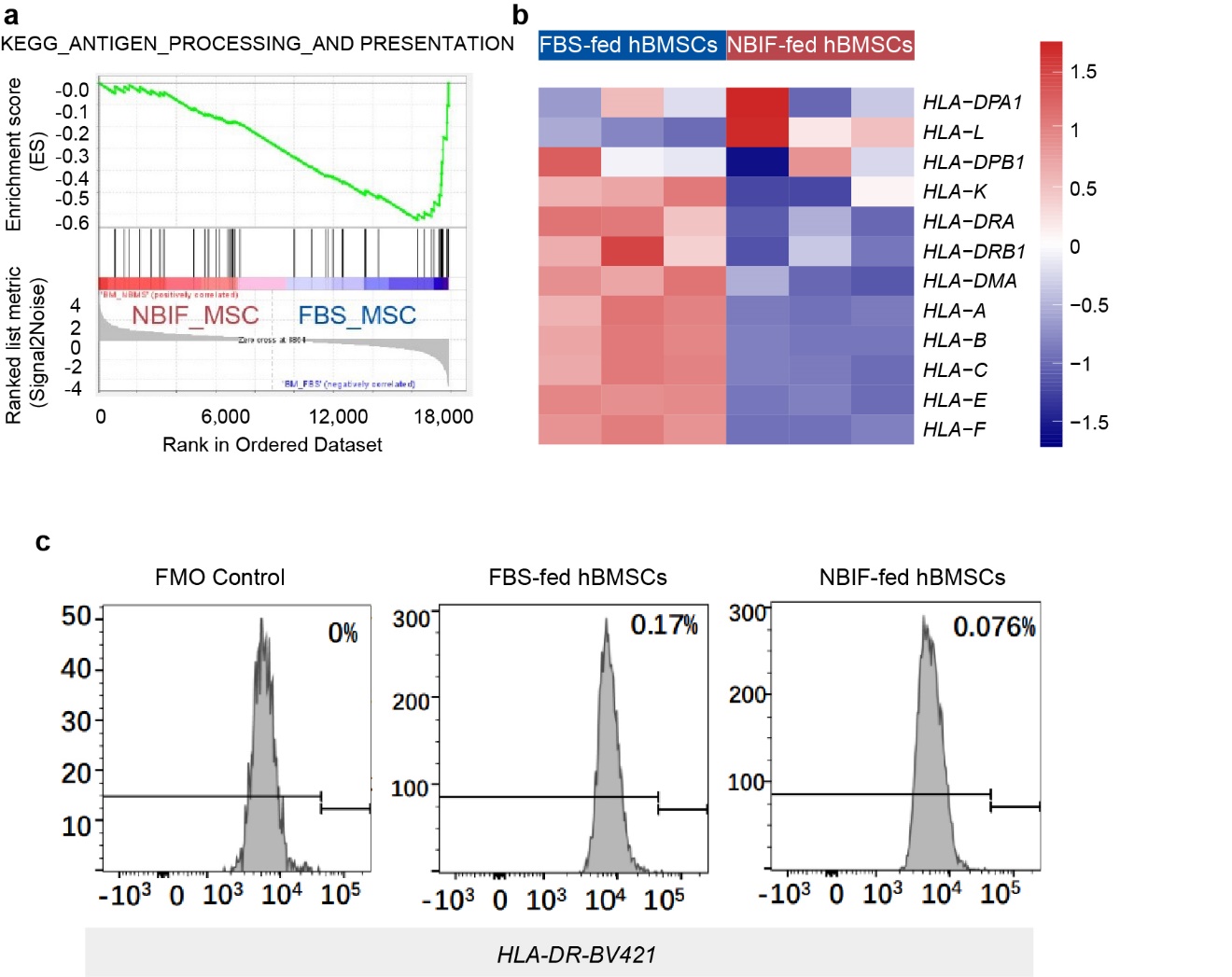


**Fig. S4** NBIF-primed hBMSCs express lower level of immunogenicity-related genes. **a** Gene Set Enrichment Analysis (GSEA) revealed significant enrichment of the KEGG Antigen Processing and Presentation pathway in NBIF-fed hBMSCs. **b** Heatmap displaying relative expression levels of major histocompatibility complexes (MHC) genes in hBMSCs primed with FBS or NBIF. **c** Flow cytometry validates the expression of HLA-DR in cells with indicated treatment. FMO control: Fluorescence Minus One Control.

**Fig. S5**


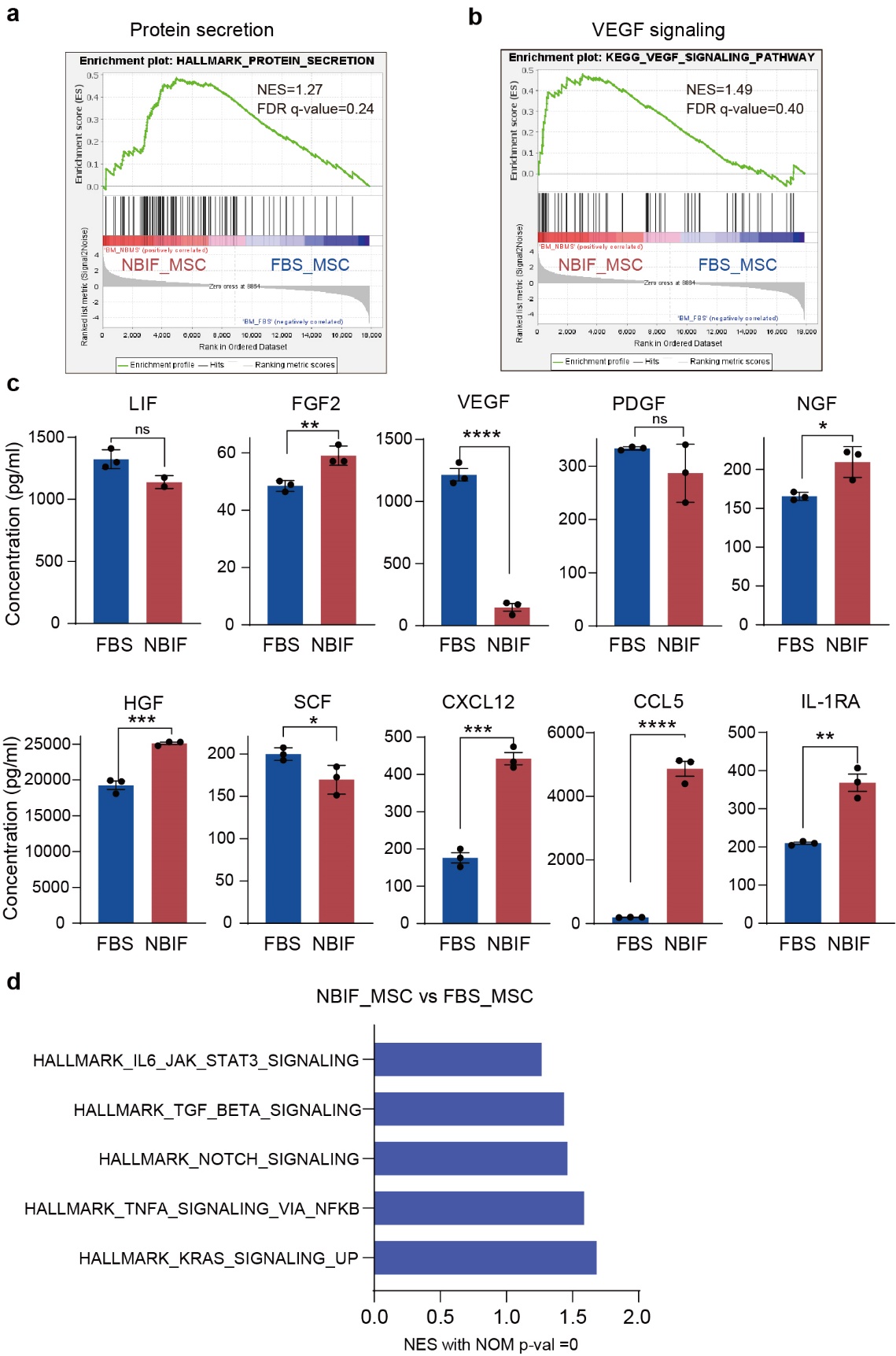


**Fig. S5** Cytokine array results for NBIF-primed hBMSCs. **a, b** Gene Set Enrichment Analysis (GSEA) revealed significant enrichment of the KEGG_Hallmark Protein Secretion pathway (**a**) and the KEGG Hallmark VEGF Signaling pathway (**b**) in NBIF-fed hBMSCs. **c** Luminex Multiplex analysis results for conditioned supernatants in hBMSCs cultured in indicated conditions. The supernatant was collected after removal of the conditioned medium and additional 24-h culture in fresh DMEM/F-12 containing 1% FBS. Data were expressed as mean ± standard error of the mean (SEM) across three replicates for each group. P-values were obtained from an unpaired *t*-test; ns, not significant, *p<0.05, **p<0.01, ***p<0.001, ****p<0.0001. **d** Gene Set Enrichment Analysis (GSEA) revealed significant enrichment of indicated signaling pathways in NBIF-fed hBMSCs. Pathways with normalized p value of 0 were shown.

**Fig. S6**

**
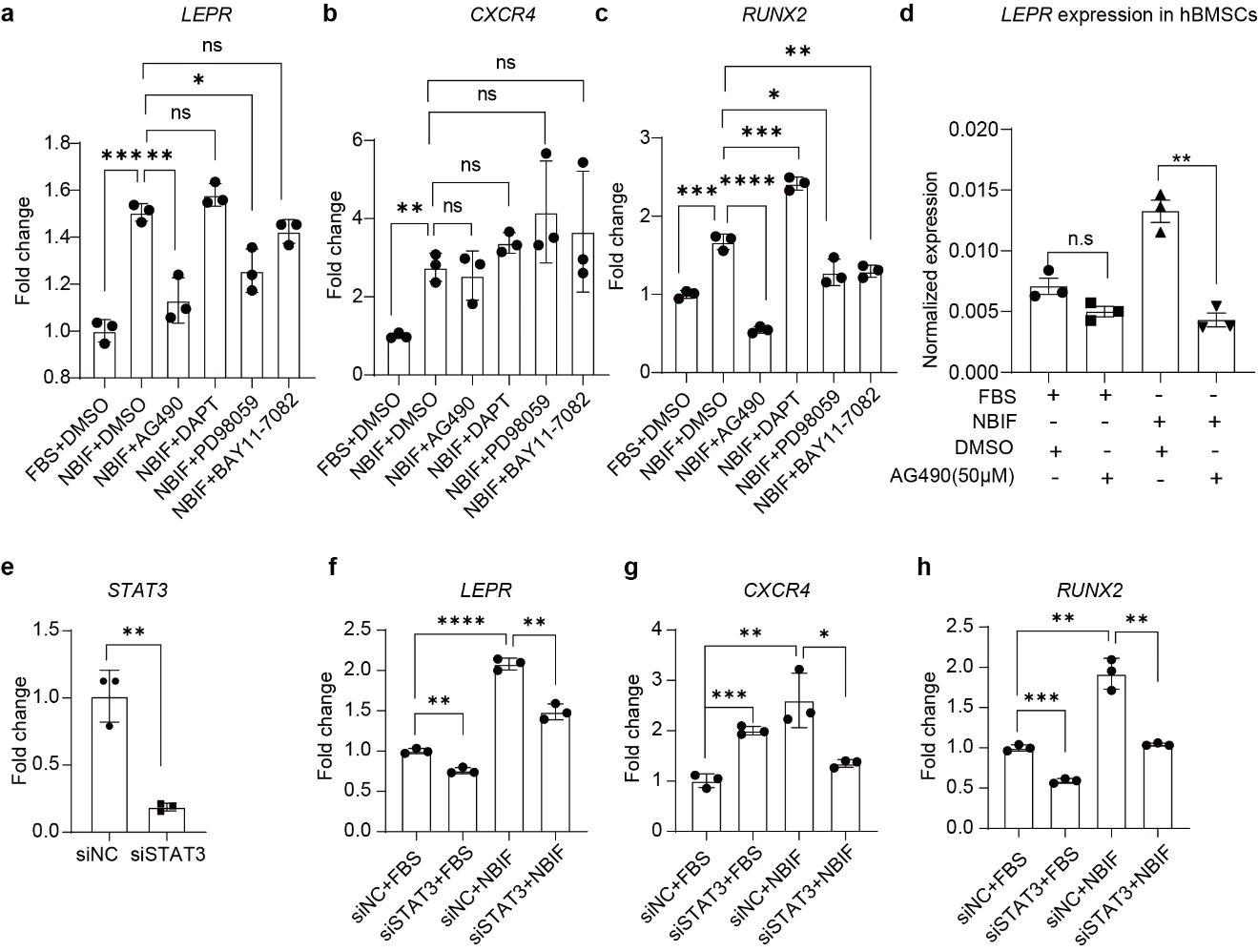
**

**Fig. S6** NBIF promotes the expression of *LEPR*, *RUNX2* and *CXCR4* via STAT3. **a-c** The effects of different inhibitors on NBIF-induced upregulation of *LEPR* (**a**), *CXCR4* (**b**) and *RUNX2* (**c**). **d** The effect of a selective STAT3 inhibitor on *LEPR* expression (normalized to *GAPDH* expression) in hBMSCs. **e** Detection of *STAT3* siRNA knockdown efficiency by qRT-PCR. **f-h** Effects of *STAT3* siRNA treatment on the expression of *LEPR*, *RUNX2* and *CXCR4* in the indicated groups. Data were expressed as mean ± standard error of the mean (SEM) across three replicates per group. P-values were obtained from an unpaired *t*-test; ns, not significant, * p ≤ 0.05, ** p ≤ 0.01, *** p ≤ 0.001, **** p ≤ 0.0001.

**Fig. S7**


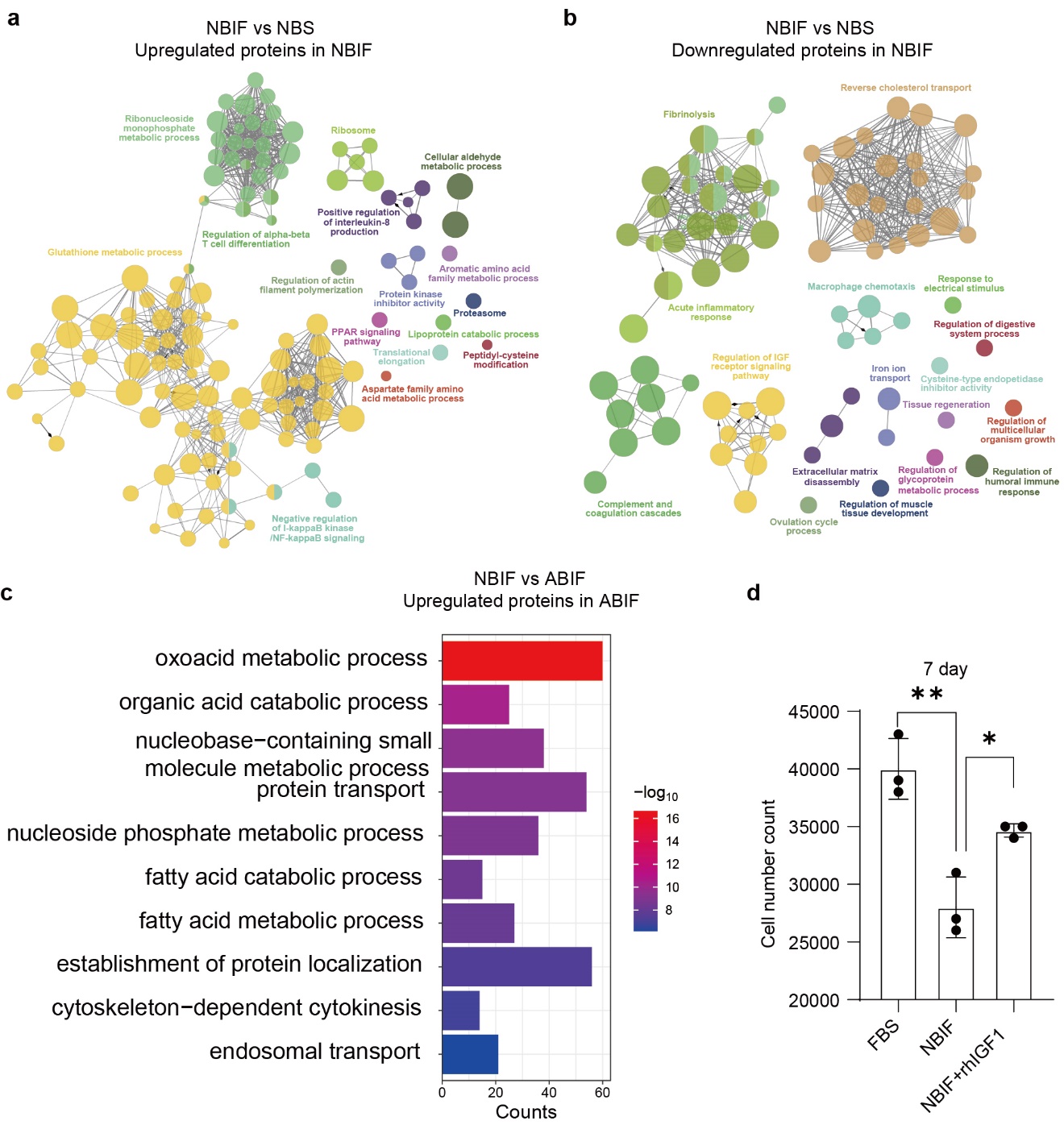


**Fig. S7** Enriched Gene Ontology (GO) networks of proteins in NBIF and ABIF. **a, b** GO analyses of upregulated (**a**) and downregulated (**b**) proteins in NBIF compared to in NBS. Networks were functionally grouped based on GO terms, Kyoto Encyclopedia of Genes and Genomes (KEGG), Reactome, and WikiPathways, with terms as nodes linked according to their κ score level (≥0.3). Only the most significant term in each group was depicted in distinct colors using ClueGO plugins in Cytoscape. **c** Enriched Gene Ontology of Biological Process (GOBP) of proteins in ABIF. The top 10 enriched biological processes were presented. **d** quantification of total cell number after 7 days’ culture under indicated condition. 5×10^3^ cells were seeded in 6 well plate and supplied with indicated medium for 7 days. Data were expressed as mean ± standard error of the mean (SEM) across three replicates per group. P-values were obtained from an unpaired *t*-test; ns, not significant, * p ≤ 0.05, ** p ≤ 0.01.

**Fig. S8**


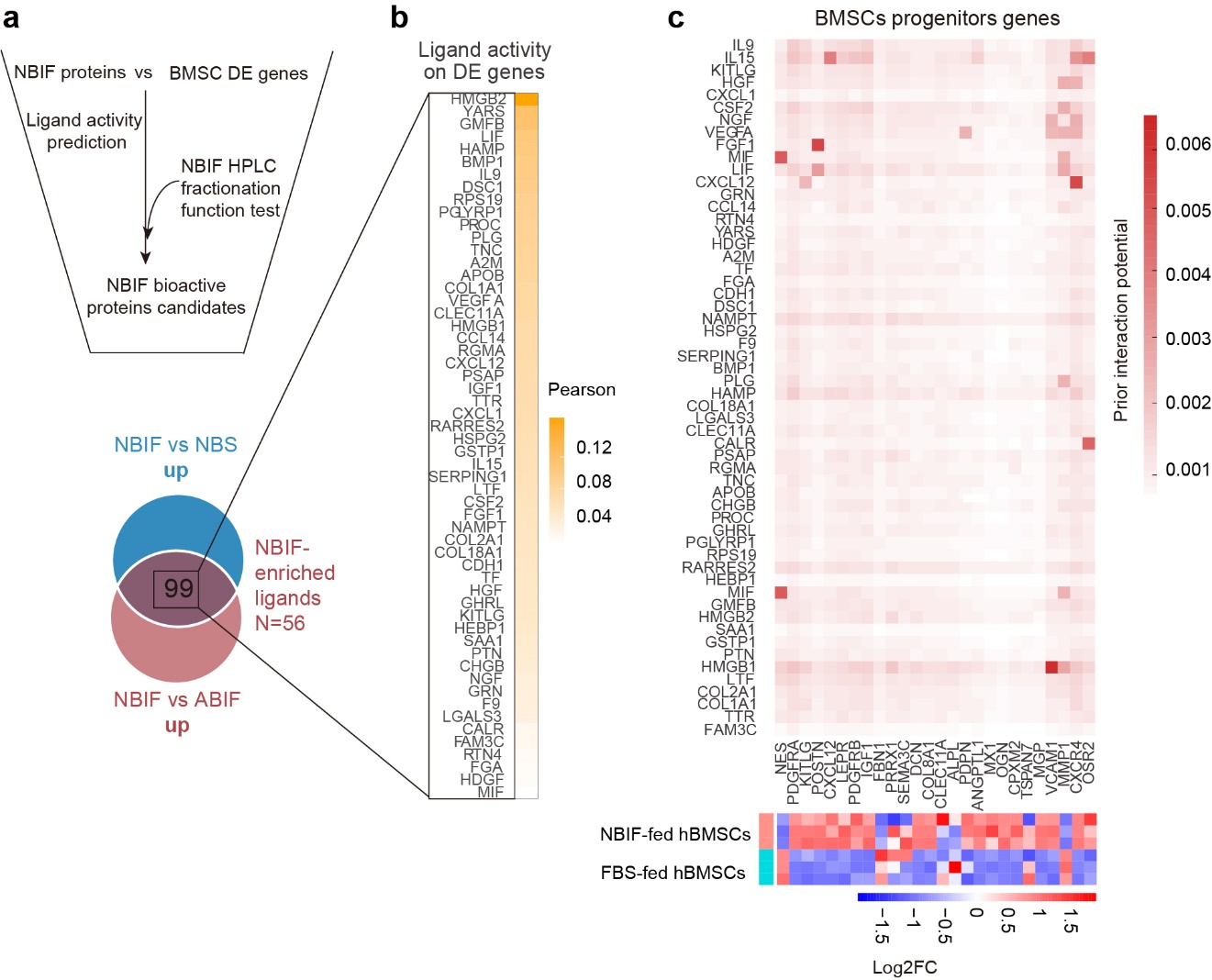


**Fig. S8** Identification of bioactive proteins from NBIF. **a** Strategy to identify bioactive proteins from NBIF. **b.** Overlap of the enriched proteins in the indicated samples. NBIF-enriched 99 proteins identified as ligand were used to analyze ligand activity on upregulated genes in NBIF-primed hBMSCs. **c** Protein interaction potential of NBIF-enriched proteins and hBMSC progenitor genes. Upper panel: 56 of 99 NBIF-enriched proteins identified as ligands in the database were used to analyze ligand activity on hBMSC progenitor genes in NBIF-primed hBMSCs by NicheNet. Lower panel: heatmap displaying relative expression levels of hBMSC progenitor genes in indicated cell groups.

**Fig. S9**


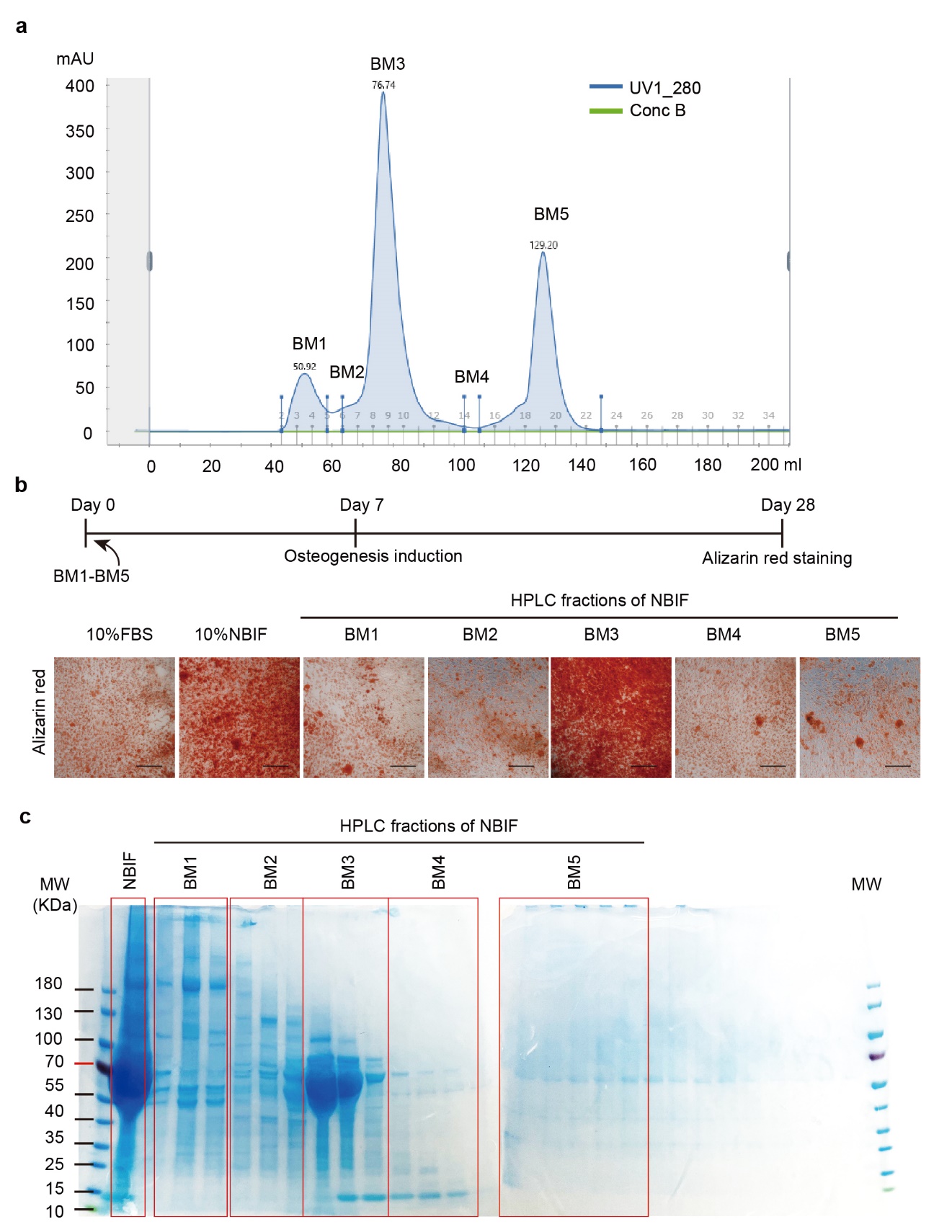


**Fig. S9** The effect of NBIF fractions on hBMSCs osteogenic potential. **a** Size-Exclusion Chromatography for the Analysis of NBIF. Flow-through proteins were detected at UV 280nm. The NBIF fractions BM1 to BM5 were shown in the graph. **b** Experiment design to study the effect of NBIF fractions on hBMSCs osteogenic potential (upper panel) and representative Alizarin reed staining images of hBMSCs (lower panel). Scale bar, 100 μm. **c** SDS-PAGE images of HPLC fractions BM1 to BM5 with molecular weights indicated.

**Fig. S10**

**
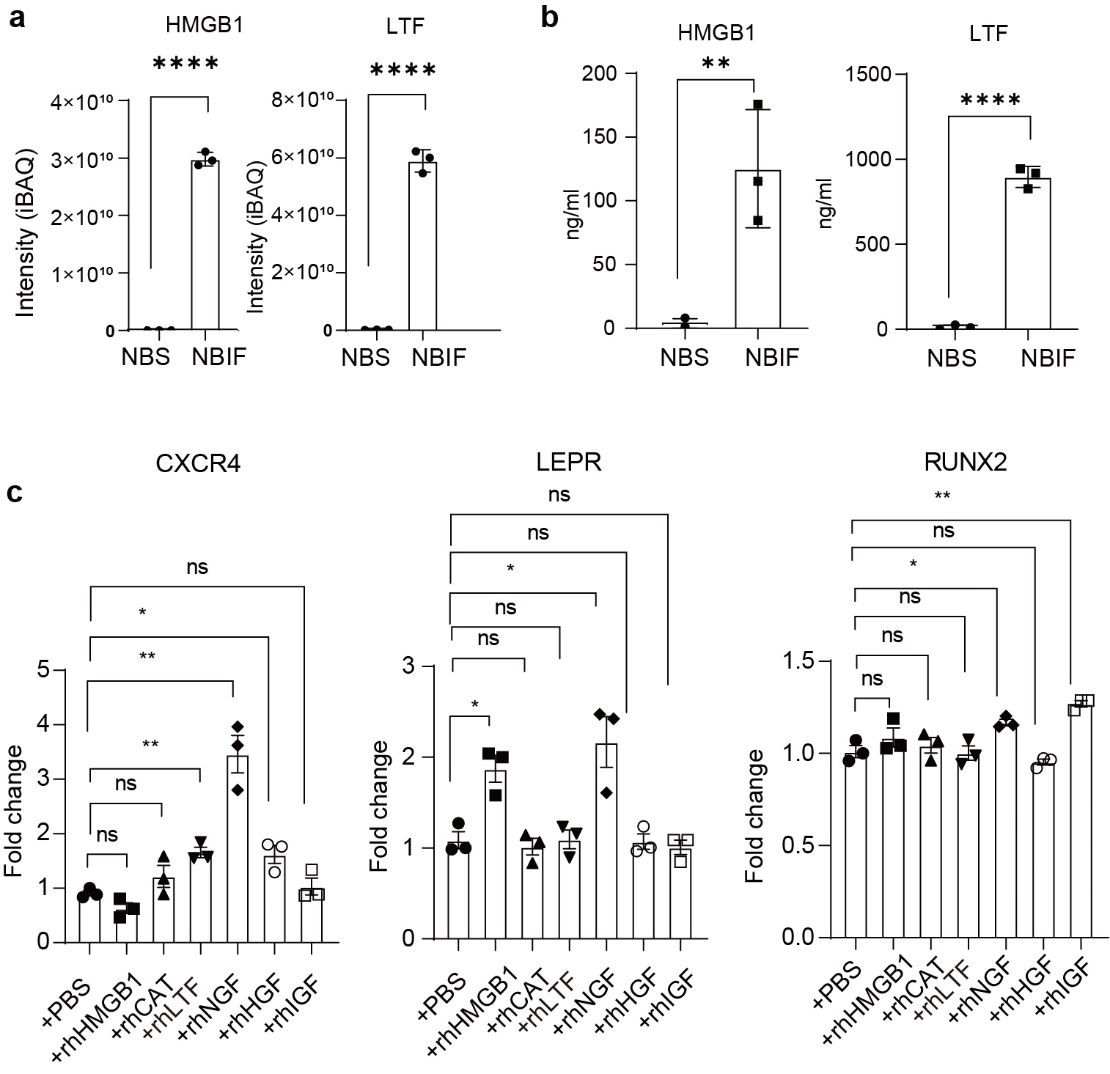
**

**Fig. S10** Effects of candidate proteins on the expression of *LEPR*, *RUNX2* and *CXCR4*. **a** Expression levels of HMGB1 and LTF in NBIF compared to NBS determined by mass spectrometry. **b** Quantification of HMGB1 and LTF protein level in NBIF by ELISA. **c** The effects of individual candidate proteins on the expression of *LEPR*, *RUNX2* and *CXCR4*. Candidate proteins at a concentration of 100 ng/ml were added to the culture medium along with FBS for 7 days, and the expression levels of the indicated genes were quantified by qRT-PCR. Data were expressed as mean ± standard error of the mean (SEM) across three replicates per group. P-values were obtained from an unpaired *t*-test; ns, not significant, * p ≤ 0.05, ** p ≤ 0.01, **** p ≤ 0.0001.

**Fig. S11**

**
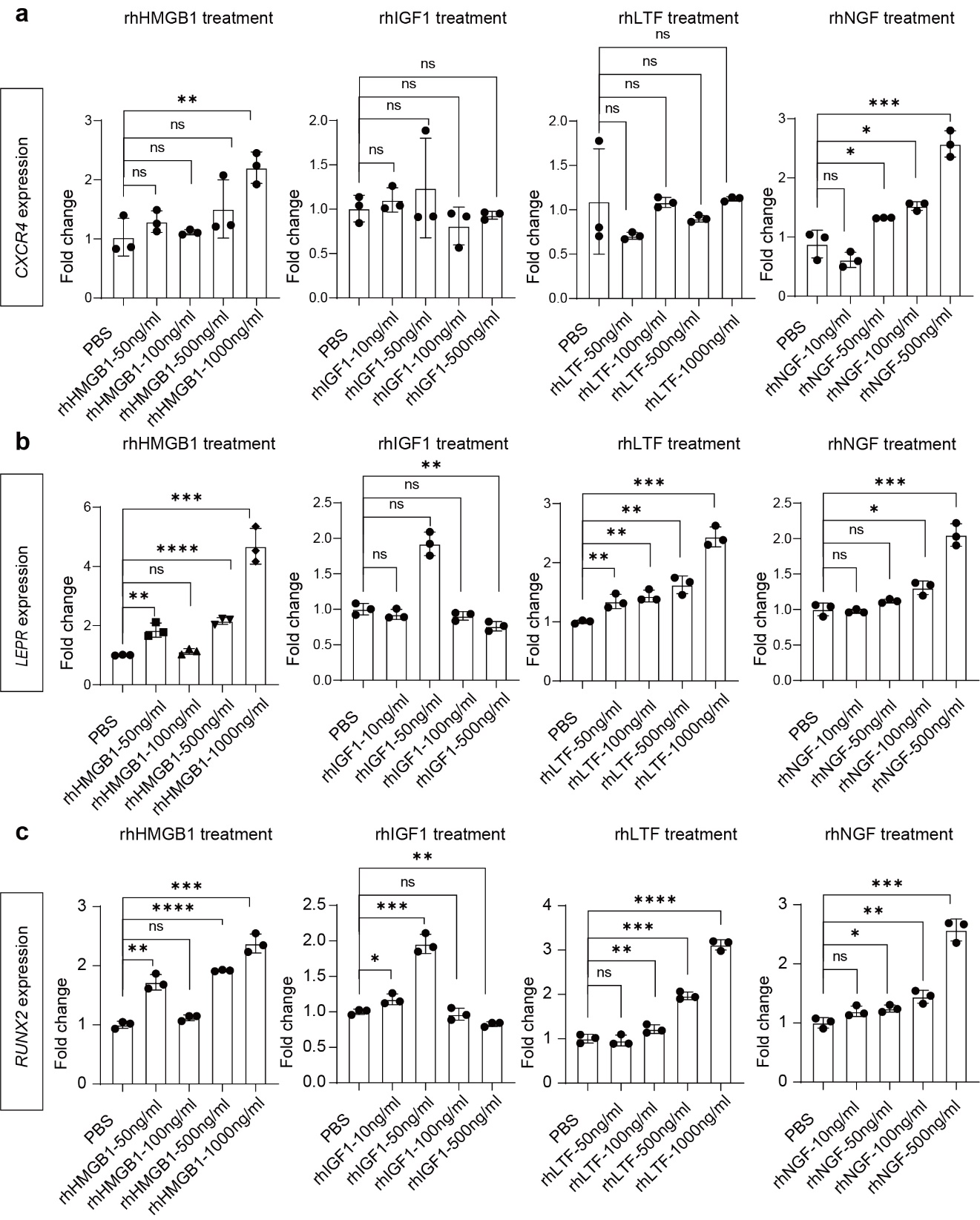
**

**Fig. S11** Dose-dependent responses of *LEPR*, *RUNX2* and *CXCR4* expression to exogenous proteins. **a-c** The expression levels of *CXCR4* (**a**), *LEPR* (**b**), *RUNX2* (**c**), and *CXCR4* using indicated proteins at different concentrations. Recombinant proteins at indicated concentration were added to the culture medium along with FBS for 7 days, and the expression levels of the indicated genes were quantified by qRT-PCR. Data were expressed as mean ± standard error of the mean (SEM) across three replicates per group. P-values were obtained from an unpaired *t*-test; ns, not significant, * p ≤ 0.05, ** p ≤ 0.01, *** p ≤ 0.001, **** p ≤ 0.0001.

**Fig. S12**


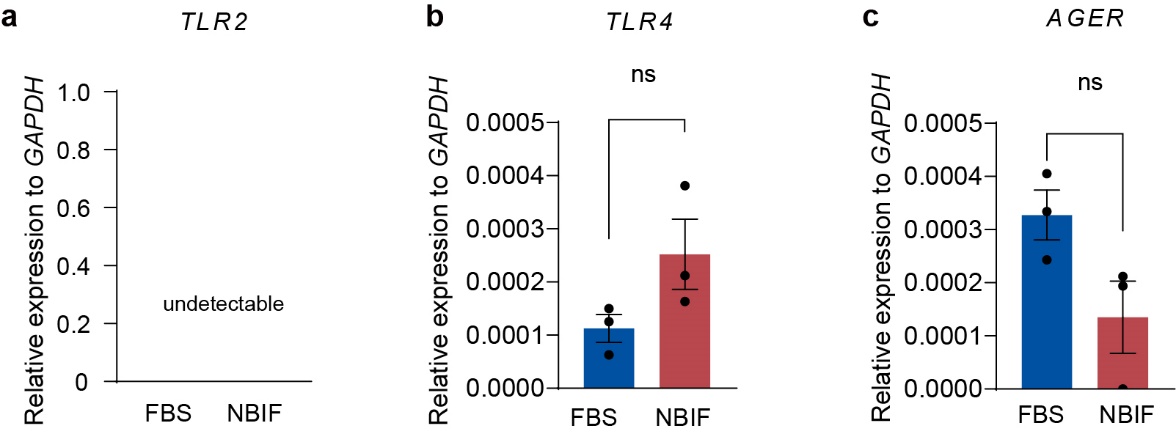


**Fig. S12** Detection of HMGB1 candidate receptor genes expression in hBMSCs. **a-c** The expression levels of the HMGB1 candidate receptors *TLR2*, *TLR4* and *AGER* in hBMSCs cultured under indicated conditions, were evaluated by quantitative RT-PCR. Data were expressed as mean ± standard error of the mean (SEM) across three replicates for each group. P-values were obtained from an unpaired *t*-test; ns, not significant.
